# Supplementary figures and images for: Acclimation of Hairless Spontaneously Hypertensive Rat to Ambient Temperature Attenuates Hypertension-Induced Pro-Arrhythmic Downregulation of Cx43 in the Left Heart Ventricle of Males
Source: Biomolecules. 2024 Nov 26;14(12):1509. doi: 10.3390/biom14121509 (PMC11674011; doi:10.3390/biom14121509)

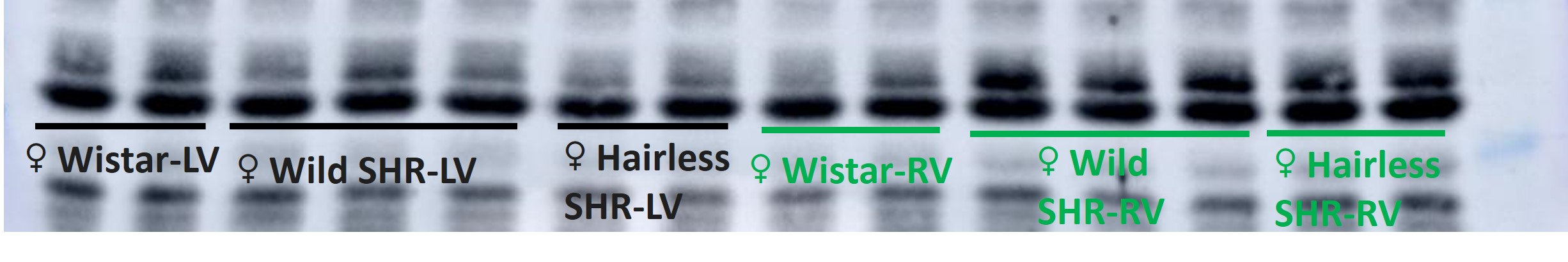

Supplement: Supplementary file 1 [file biomolecules-14-01509-s001.zip › GAPDH MMP2 FEMALE_membrane 1.jpg]

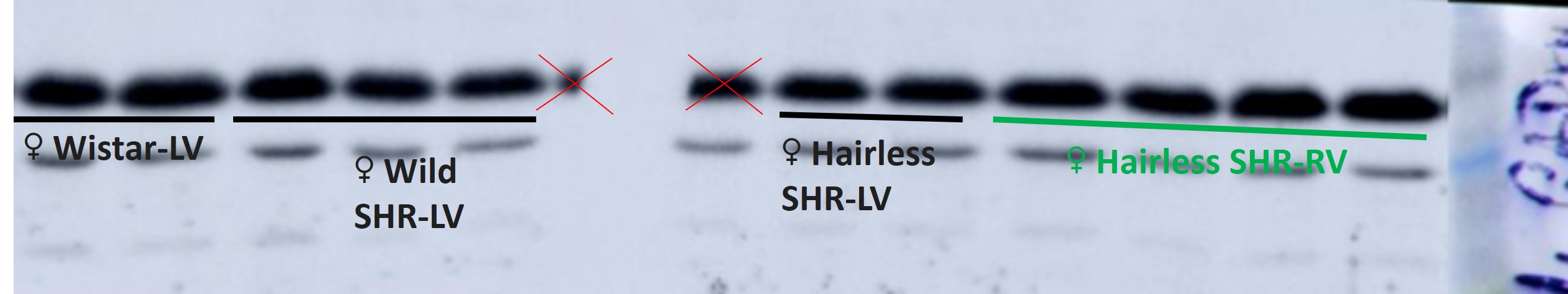

Supplement: Supplementary file 1 [file biomolecules-14-01509-s001.zip › GAPDH MMP2 FEMALE_membrane 2.jpg]

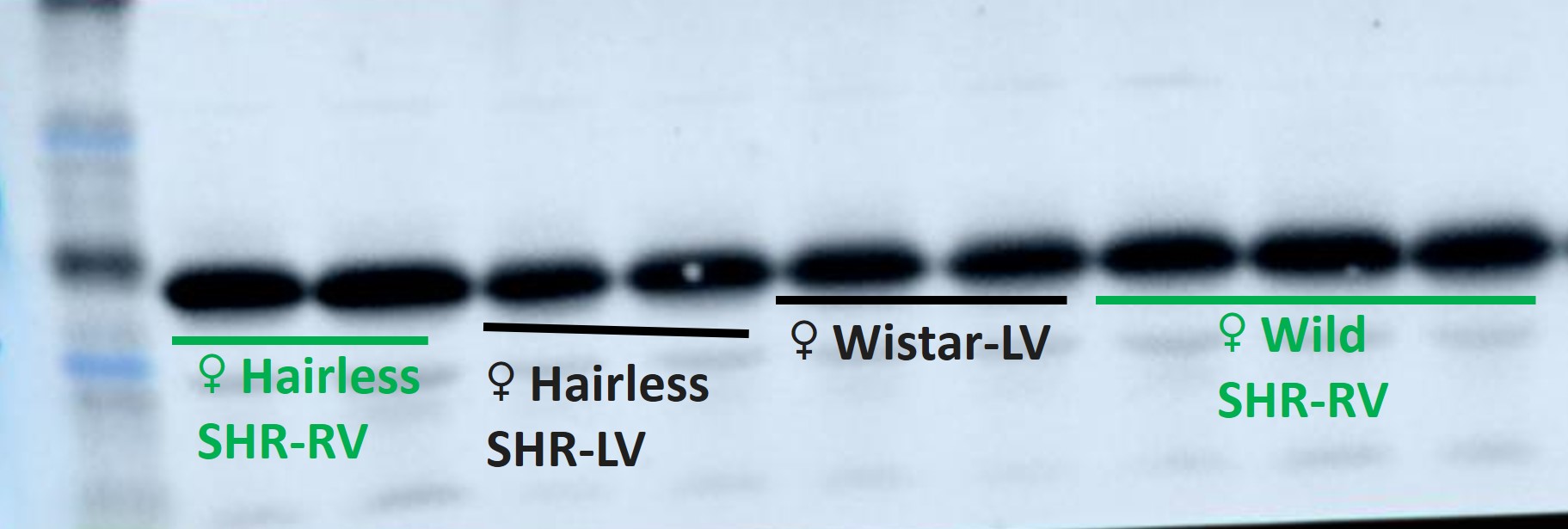

Supplement: Supplementary file 1 [file biomolecules-14-01509-s001.zip › GAPDH MMP2 FEMALE_membrane 3.jpg]

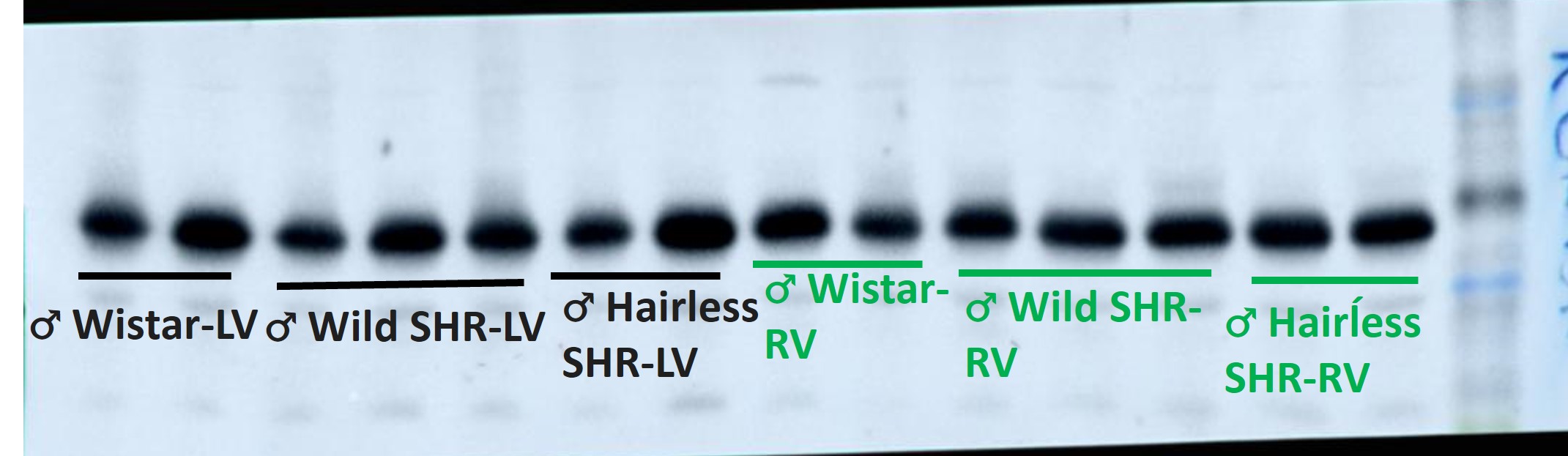

Supplement: Supplementary file 1 [file biomolecules-14-01509-s001.zip › GAPDH MMP2 MALE_membrane 1.jpg]

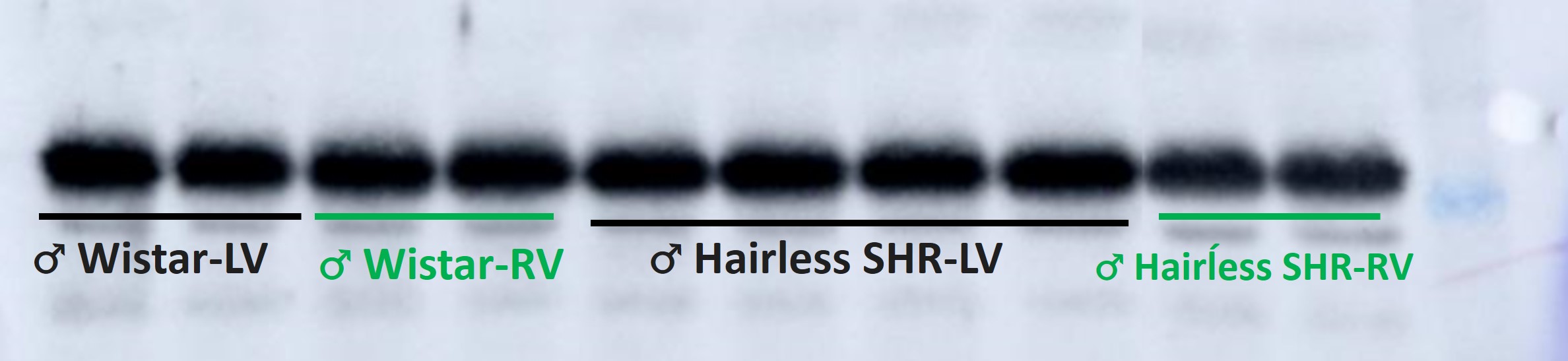

Supplement: Supplementary file 1 [file biomolecules-14-01509-s001.zip › GAPDH MMP2 MALE_membrane 2.jpg]

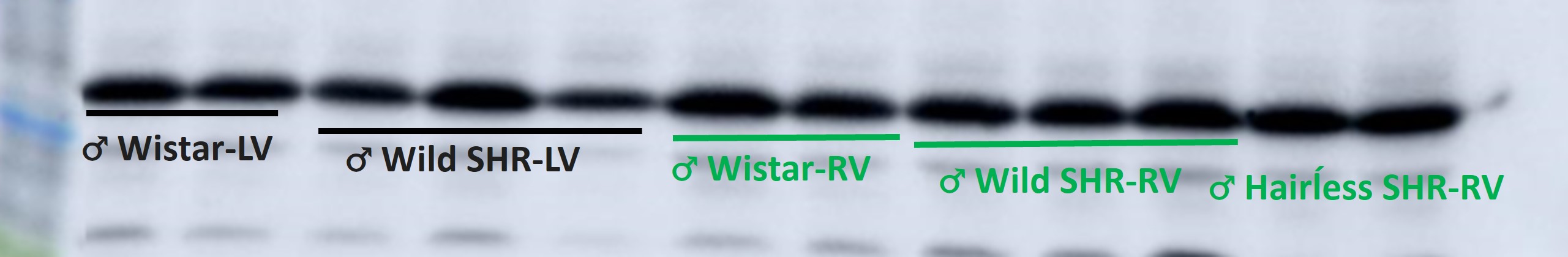

Supplement: Supplementary file 1 [file biomolecules-14-01509-s001.zip › GAPDH MMP2 MALE_membrane 3.jpg]

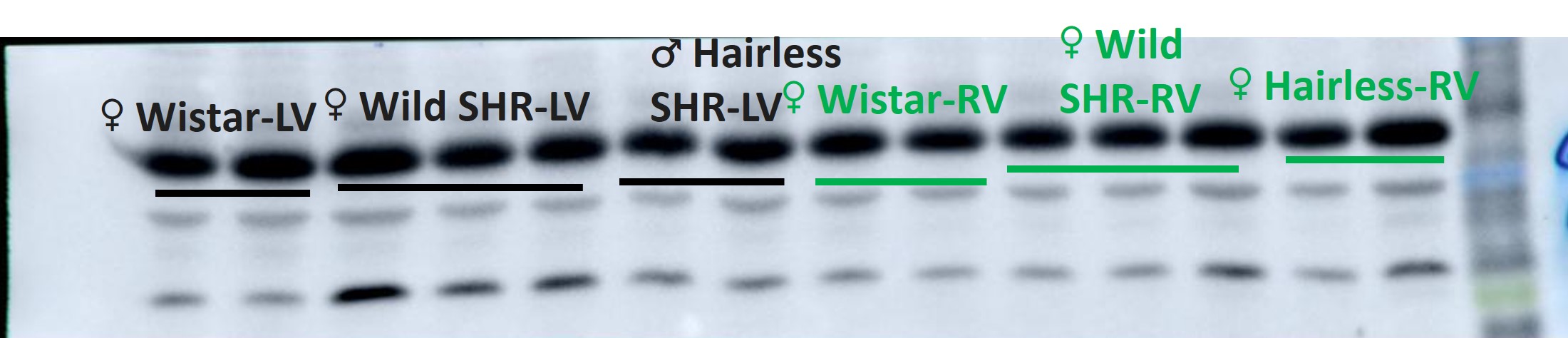

Supplement: Supplementary file 1 [file biomolecules-14-01509-s001.zip › GAPDH PKCdelta FEMALE_membrane 1.jpg]

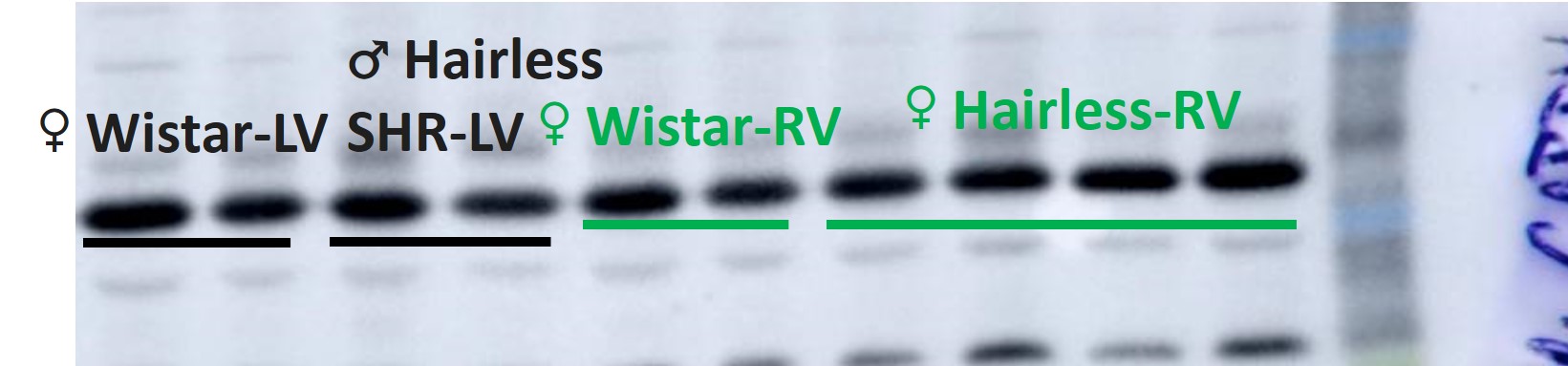

Supplement: Supplementary file 1 [file biomolecules-14-01509-s001.zip › GAPDH PKCdelta FEMALE_membrane 2.jpg]

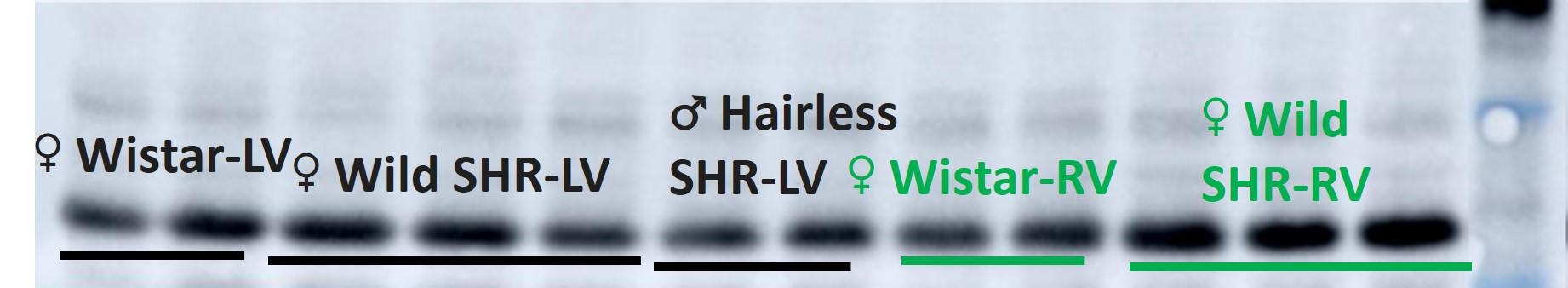

Supplement: Supplementary file 1 [file biomolecules-14-01509-s001.zip › GAPDH PKCdelta FEMALE_membrane 3.jpg]

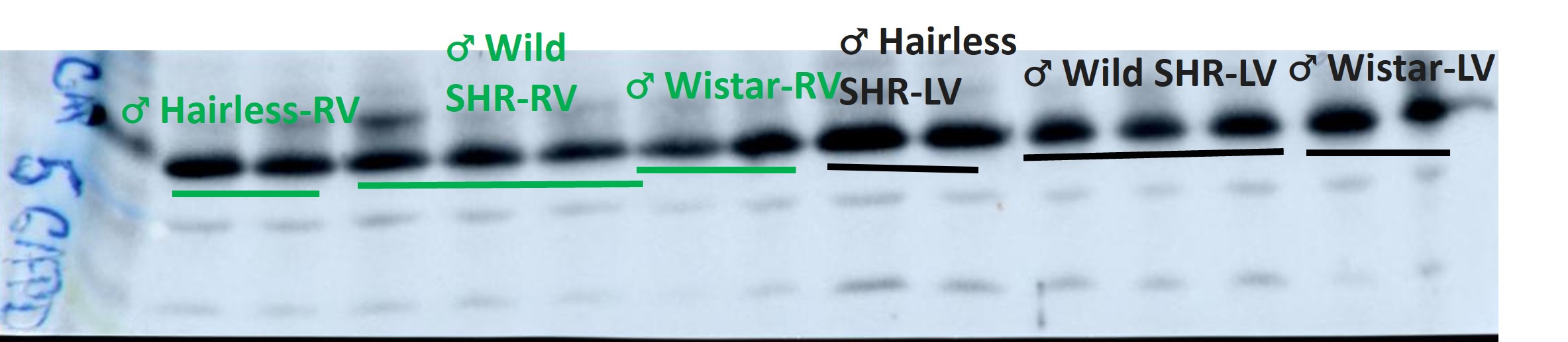

Supplement: Supplementary file 1 [file biomolecules-14-01509-s001.zip › GAPDH PKCdelta MALE_membrane 1.jpg]

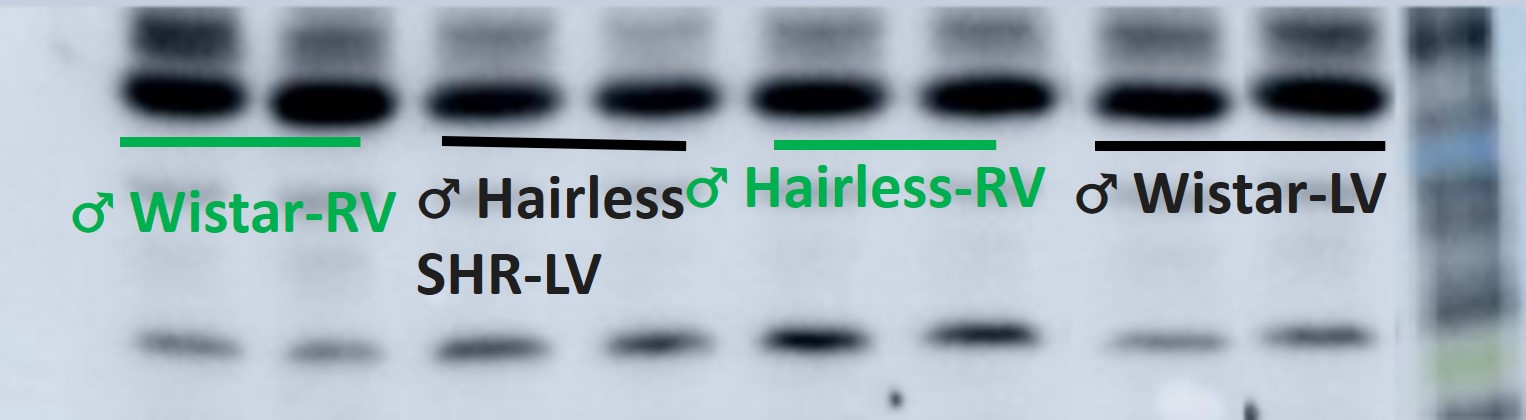

Supplement: Supplementary file 1 [file biomolecules-14-01509-s001.zip › GAPDH PKCdelta MALE_membrane 2.jpg]

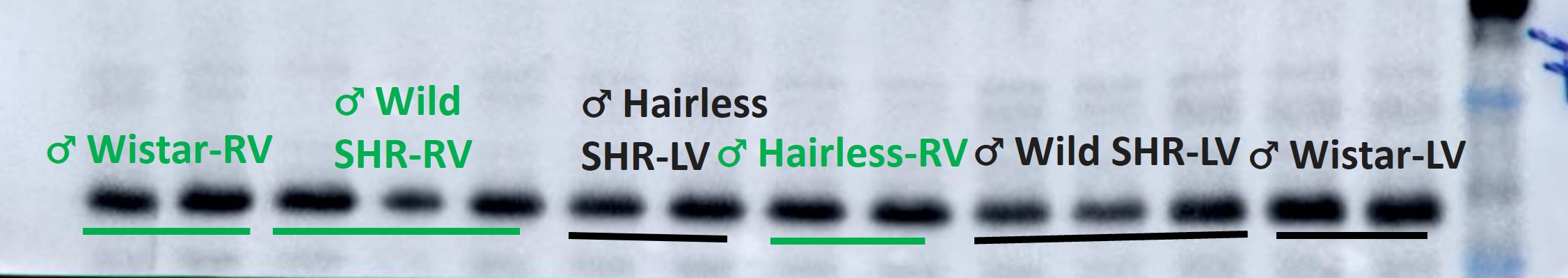

Supplement: Supplementary file 1 [file biomolecules-14-01509-s001.zip › GAPDH PKCdelta MALE_membrane 3.jpg]

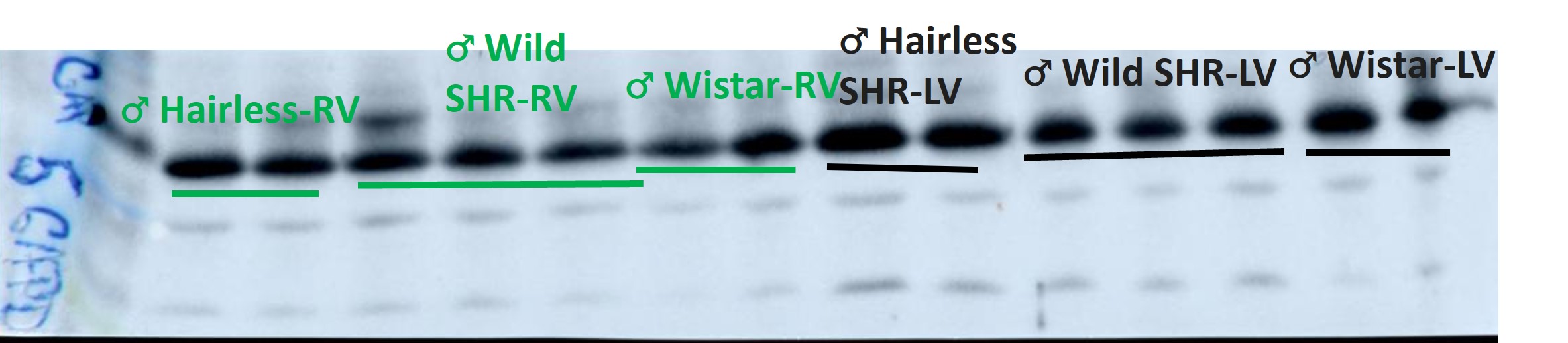

Supplement: Supplementary file 1 [file biomolecules-14-01509-s001.zip › GAPDH PKCepsilon FEMALE_membrane 1.jpg]

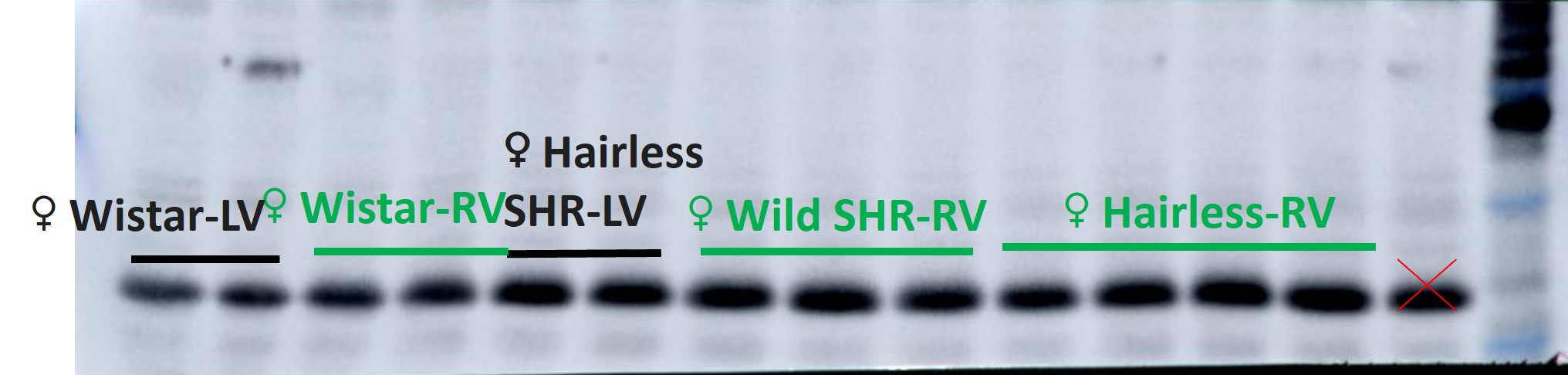

Supplement: Supplementary file 1 [file biomolecules-14-01509-s001.zip › GAPDH PKCepsilon FEMALE_membrane 2.jpg]

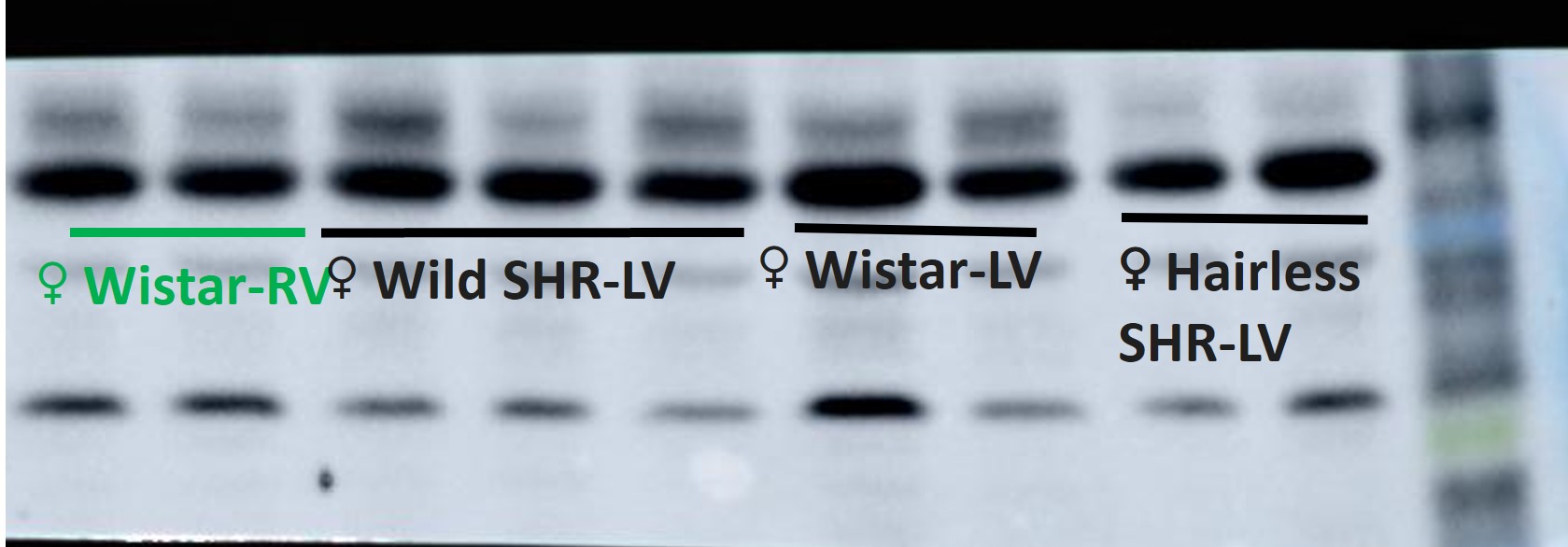

Supplement: Supplementary file 1 [file biomolecules-14-01509-s001.zip › GAPDH PKCepsilon FEMALE_membrane 3.jpg]

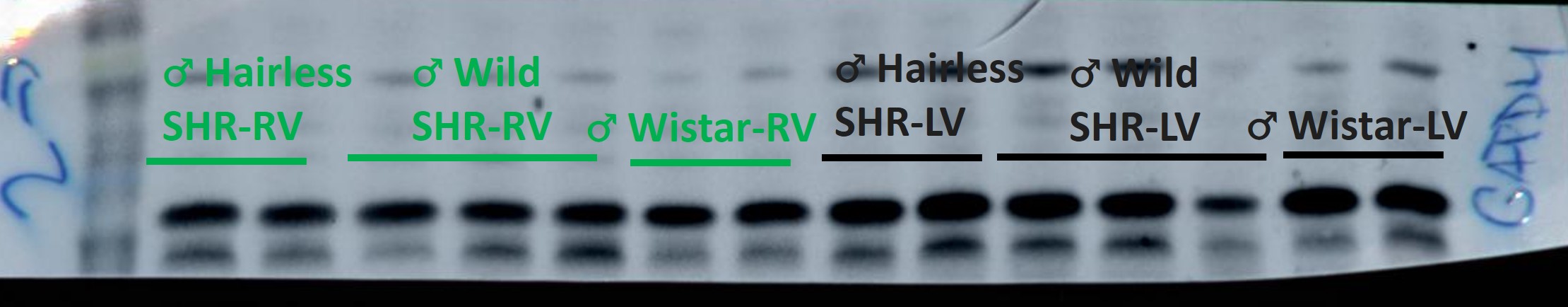

Supplement: Supplementary file 1 [file biomolecules-14-01509-s001.zip › GAPDH PKCepsilon MALE_membrane 1.jpg]

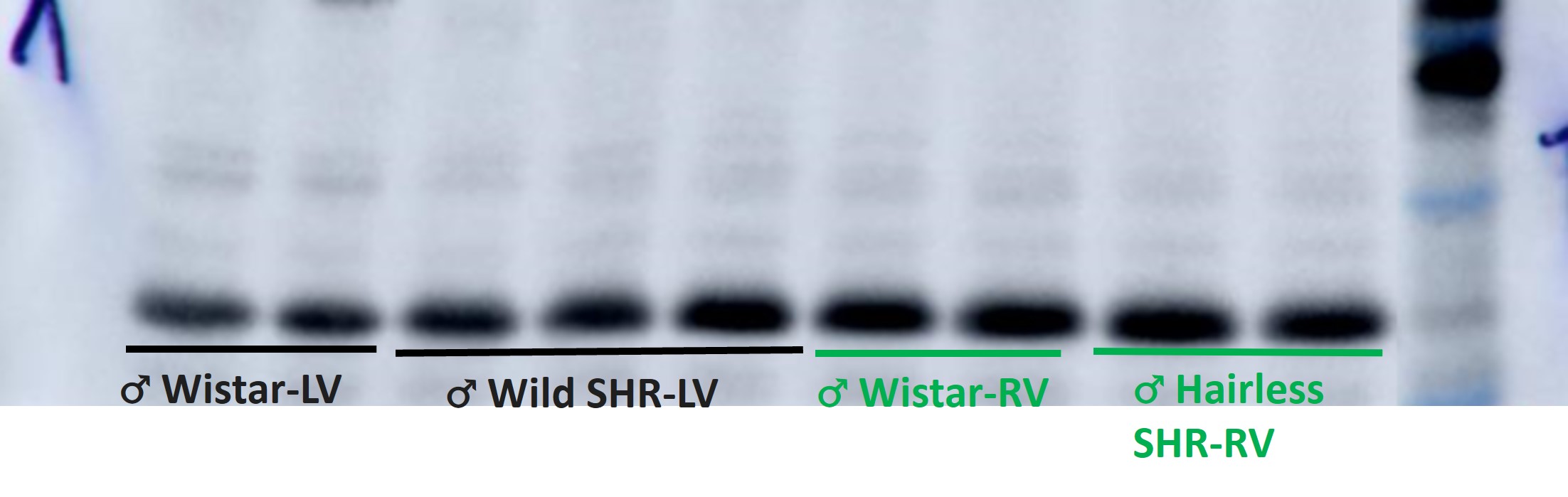

Supplement: Supplementary file 1 [file biomolecules-14-01509-s001.zip › GAPDH PKCepsilon MALE_membrane 2.jpg]

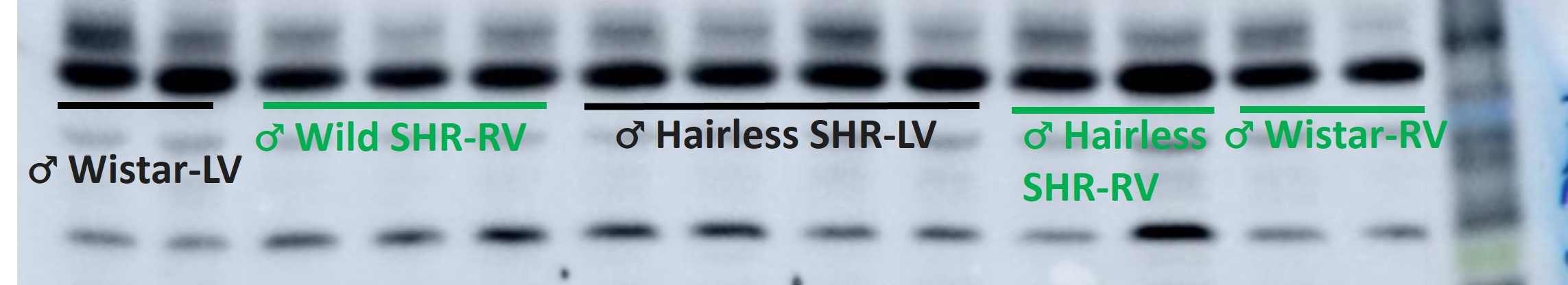

Supplement: Supplementary file 1 [file biomolecules-14-01509-s001.zip › GAPDH PKCepsilon MALE_membrane 3.jpg]

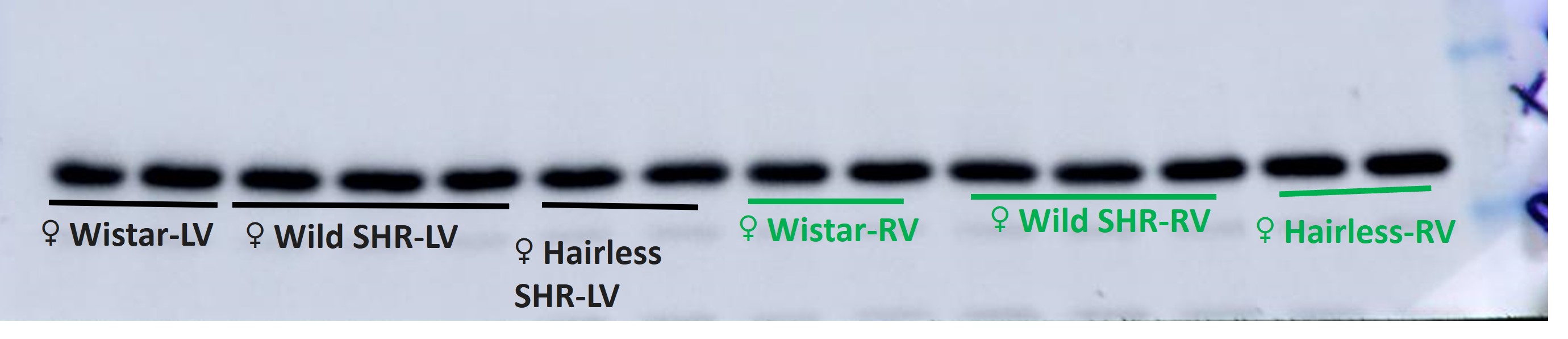

Supplement: Supplementary file 1 [file biomolecules-14-01509-s001.zip › GAPDH SMAD FEMALE_membrane 1.jpg]

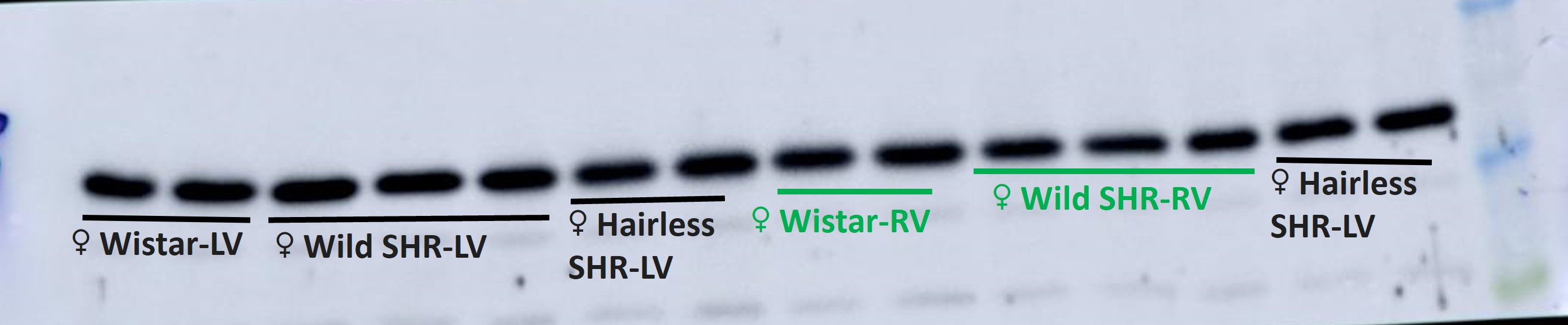

Supplement: Supplementary file 1 [file biomolecules-14-01509-s001.zip › GAPDH SMAD FEMALE_membrane 2.jpg]

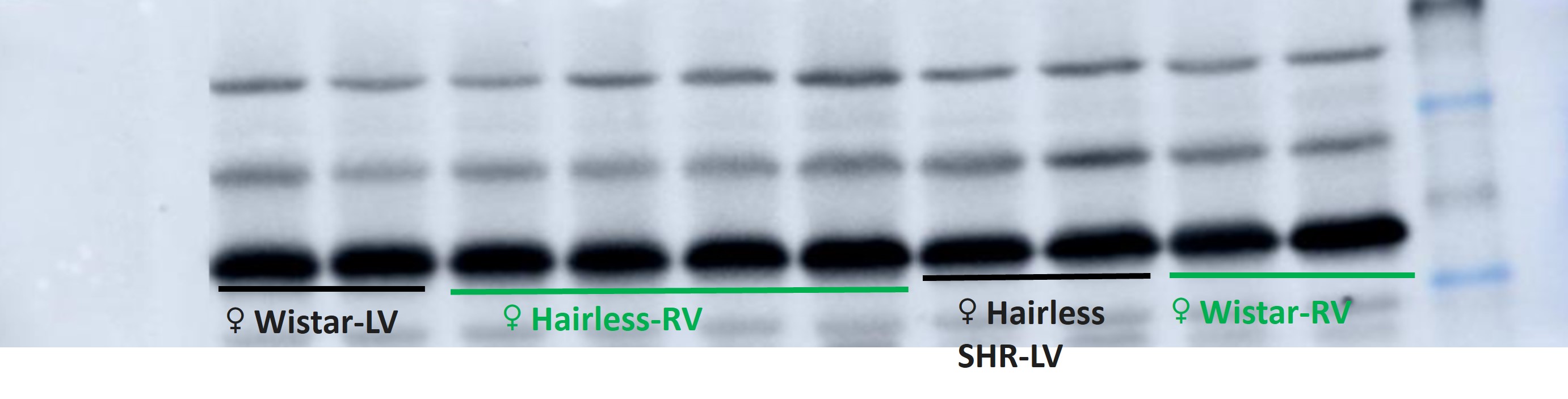

Supplement: Supplementary file 1 [file biomolecules-14-01509-s001.zip › GAPDH SMAD FEMALE_membrane 3.jpg]

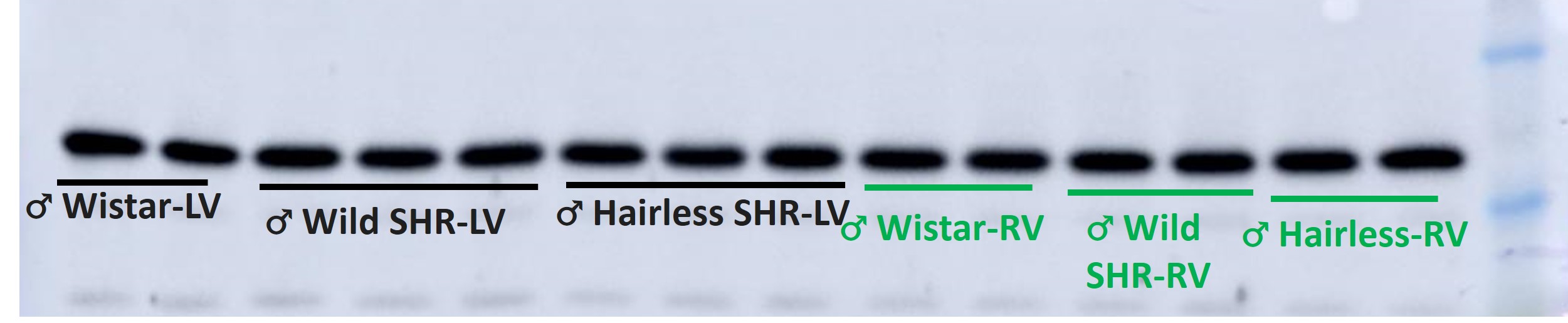

Supplement: Supplementary file 1 [file biomolecules-14-01509-s001.zip › GAPDH SMAD MALE_membrane 1.jpg]

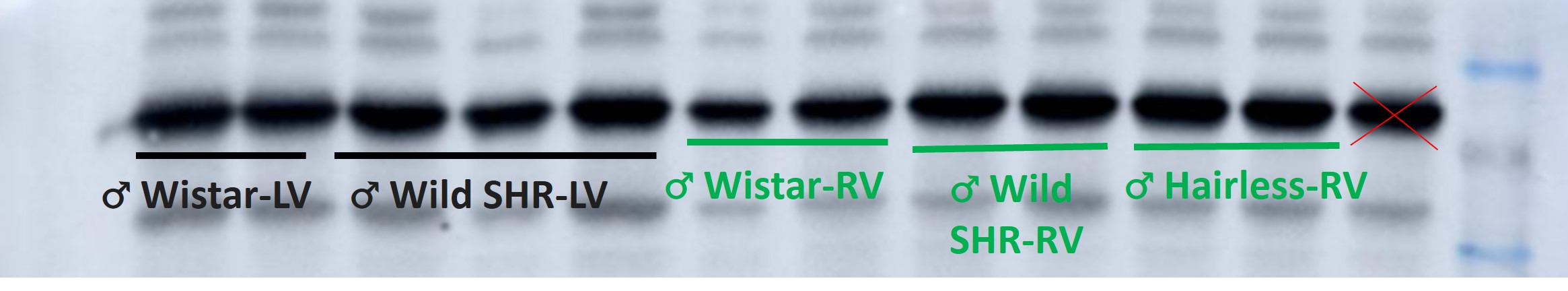

Supplement: Supplementary file 1 [file biomolecules-14-01509-s001.zip › GAPDH SMAD MALE_membrane 2.jpg]

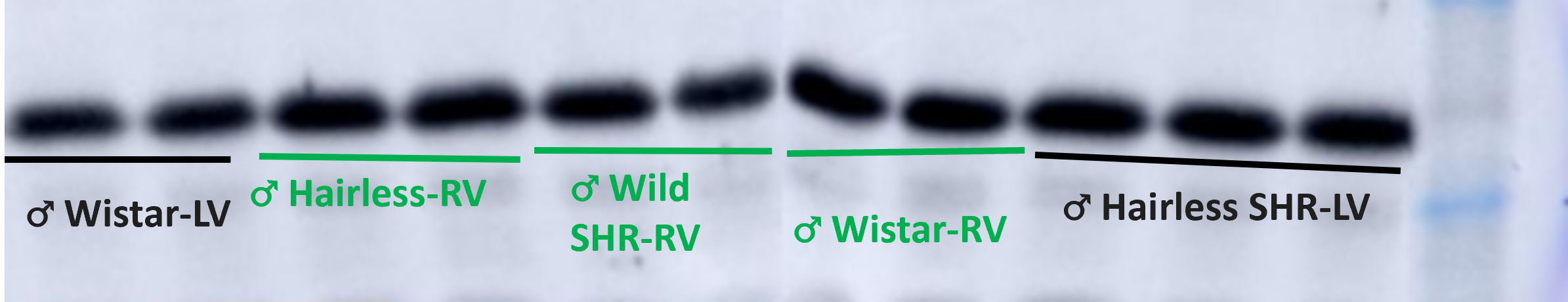

Supplement: Supplementary file 1 [file biomolecules-14-01509-s001.zip › GAPDH SMAD MALE_membrane 3.png]

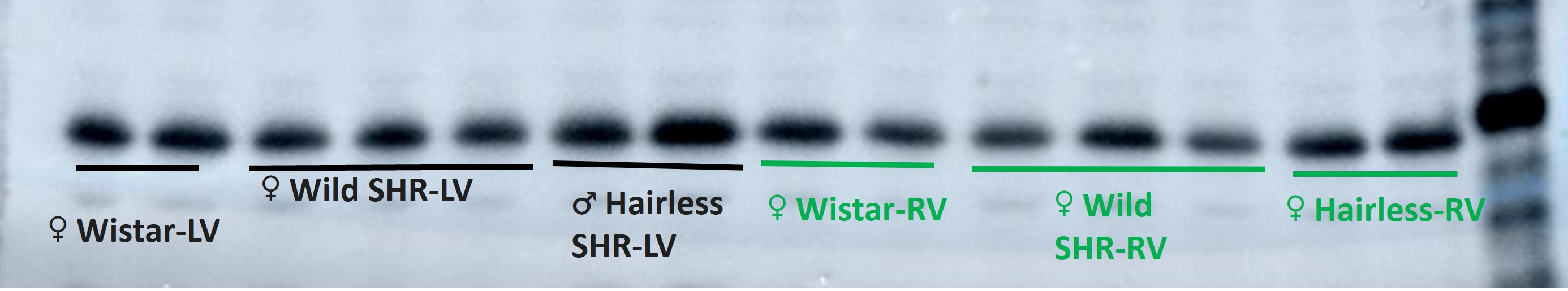

Supplement: Supplementary file 1 [file biomolecules-14-01509-s001.zip › GAPDH TGFbeta FEMALE_membrane 1.jpg]

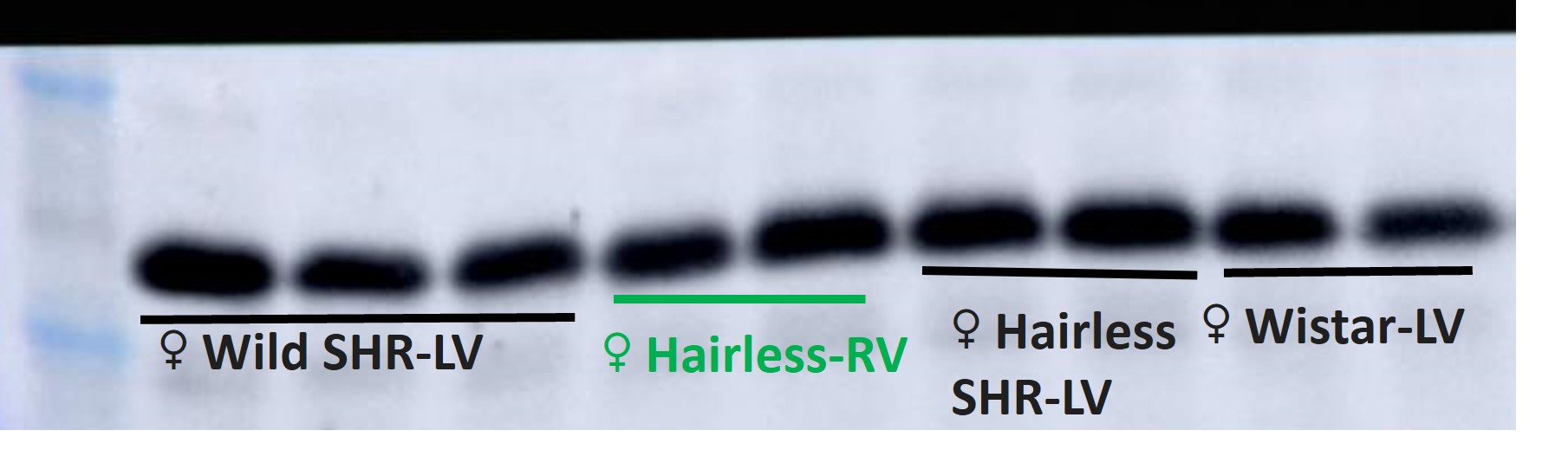

Supplement: Supplementary file 1 [file biomolecules-14-01509-s001.zip › GAPDH TGFbeta FEMALE_membrane 2.jpg]

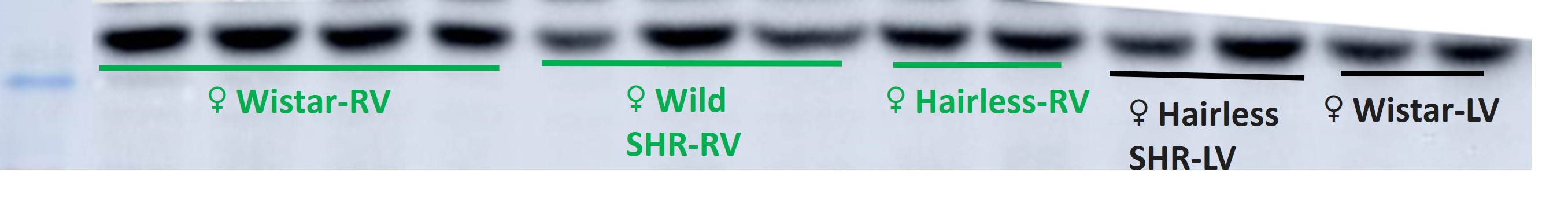

Supplement: Supplementary file 1 [file biomolecules-14-01509-s001.zip › GAPDH TGFbeta FEMALE_membrane 3.jpg]

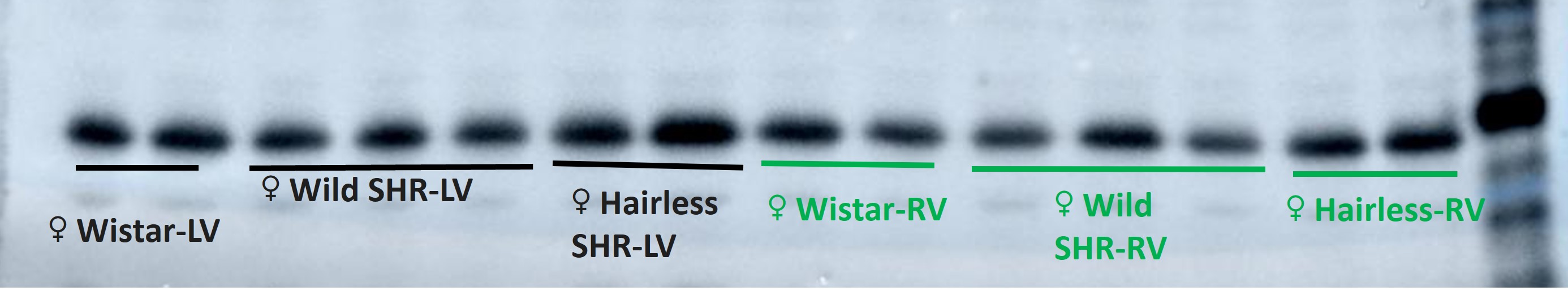

Supplement: Supplementary file 1 [file biomolecules-14-01509-s001.zip › GAPDH TGFbeta MALE_membrane 1.jpg]

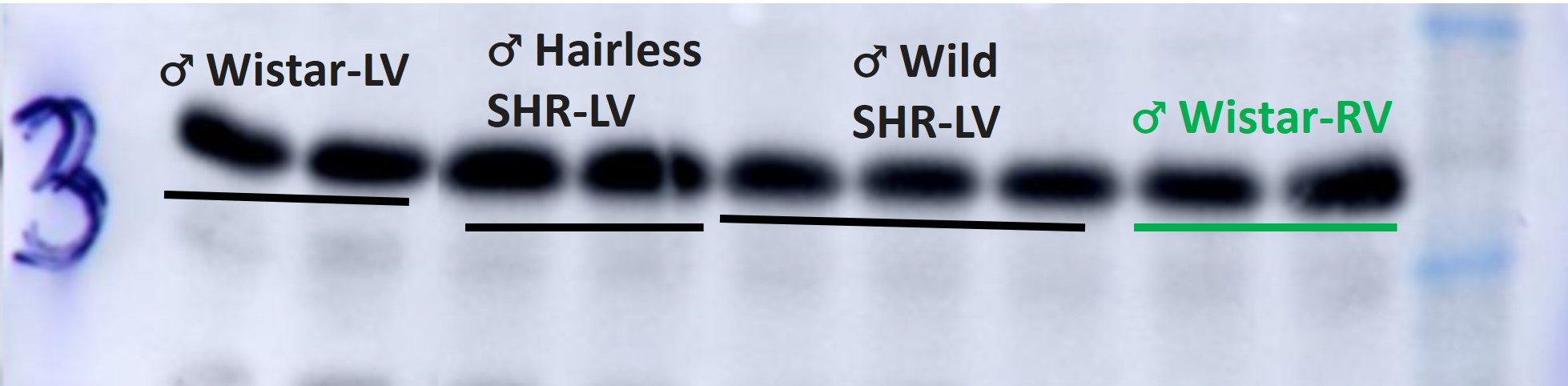

Supplement: Supplementary file 1 [file biomolecules-14-01509-s001.zip › GAPDH TGFbeta MALE_membrane 2.jpg]

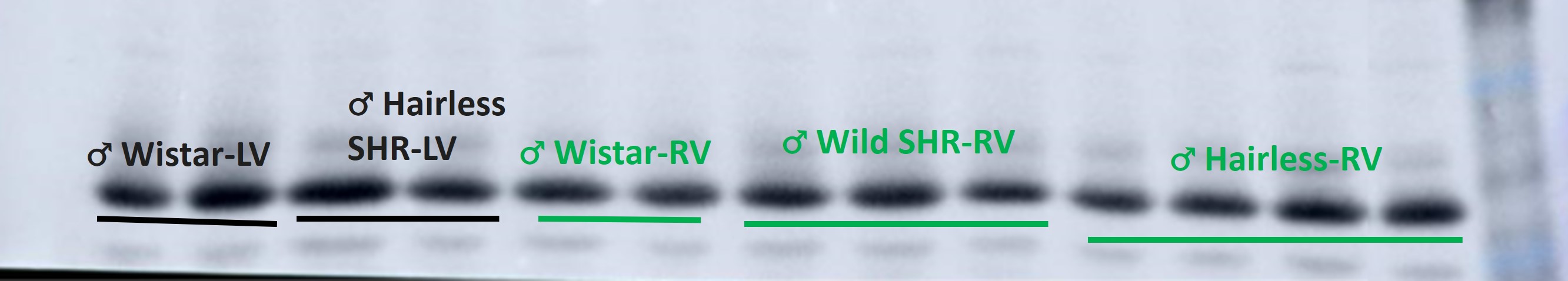

Supplement: Supplementary file 1 [file biomolecules-14-01509-s001.zip › GAPDH TGFbeta MALE_membrane 3.jpg]

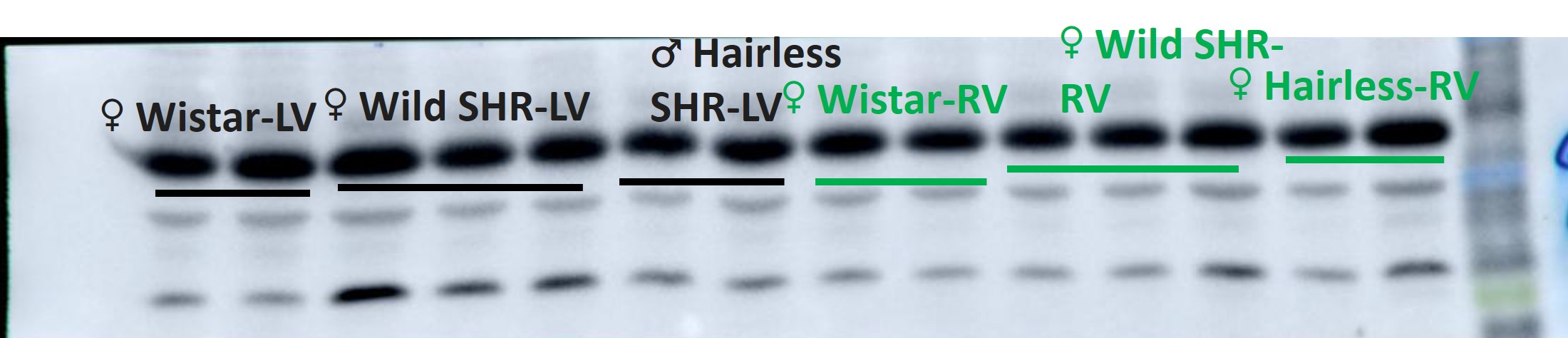

Supplement: Supplementary file 1 [file biomolecules-14-01509-s001.zip › GAPDH to p368Cx43 FEMALE_membrane 1.jpg]

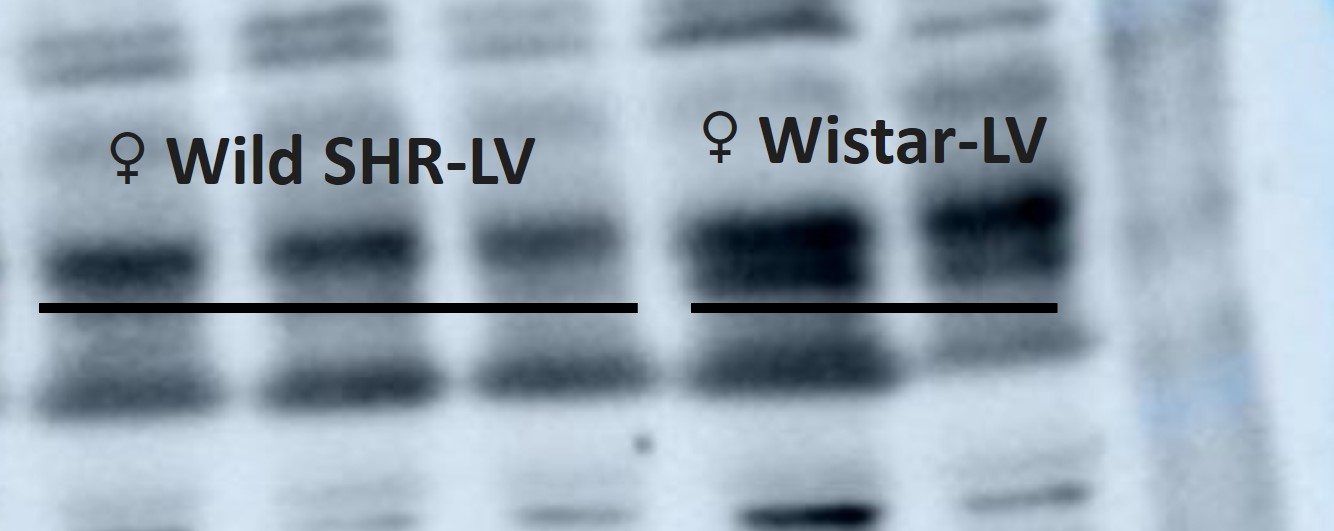

Supplement: Supplementary file 1 [file biomolecules-14-01509-s001.zip › GAPDH to p368Cx43 FEMALE_membrane 2.jpg]

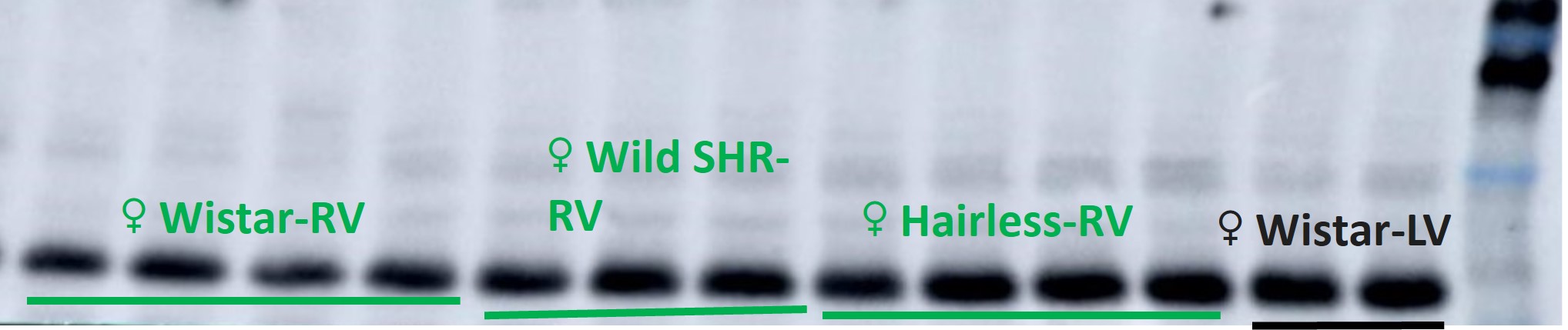

Supplement: Supplementary file 1 [file biomolecules-14-01509-s001.zip › GAPDH to p368Cx43 FEMALE_membrane 3.jpg]

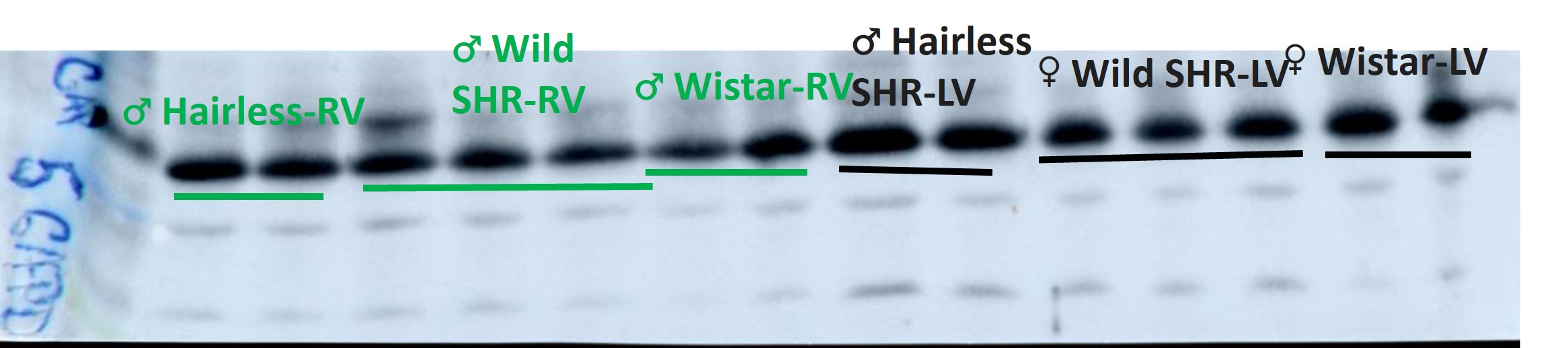

Supplement: Supplementary file 1 [file biomolecules-14-01509-s001.zip › GAPDH to p368Cx43 MALE_membrane 1.jpg]

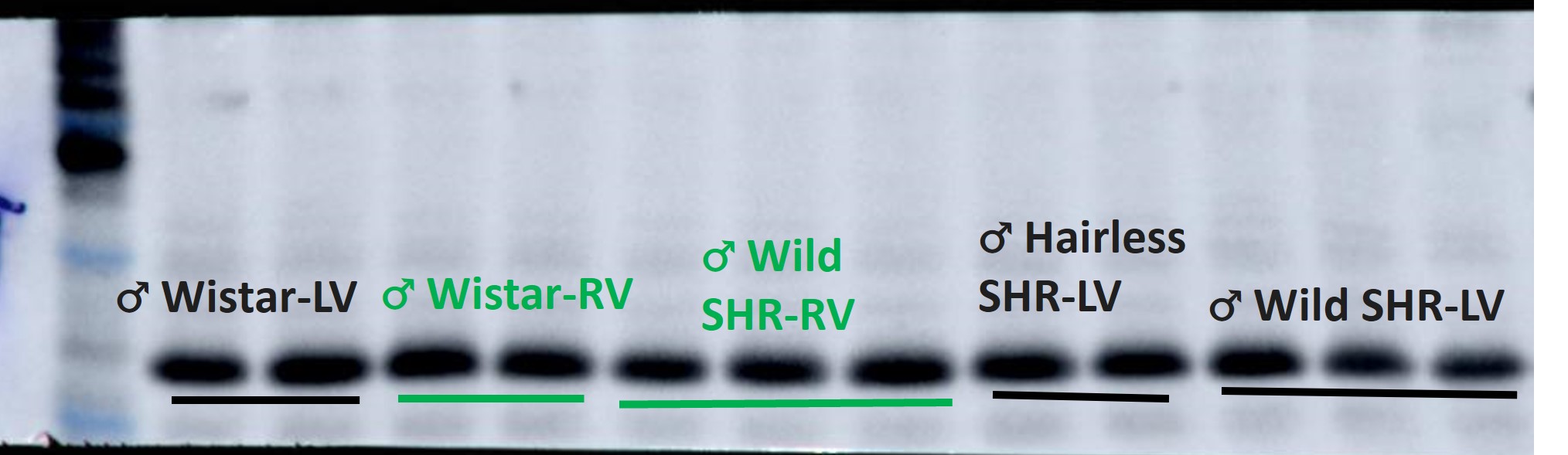

Supplement: Supplementary file 1 [file biomolecules-14-01509-s001.zip › GAPDH to p368Cx43 MALE_membrane 2.jpg]

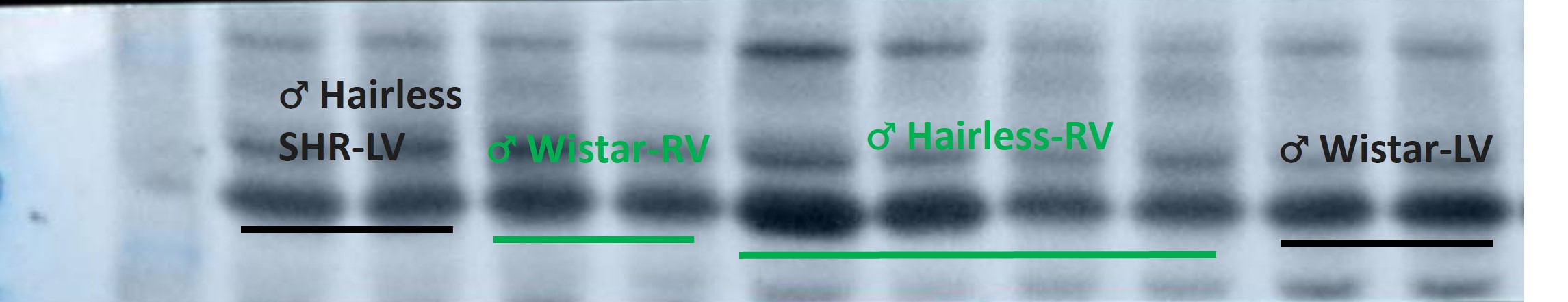

Supplement: Supplementary file 1 [file biomolecules-14-01509-s001.zip › GAPDH to p368Cx43 MALE_membrane 3.jpg]

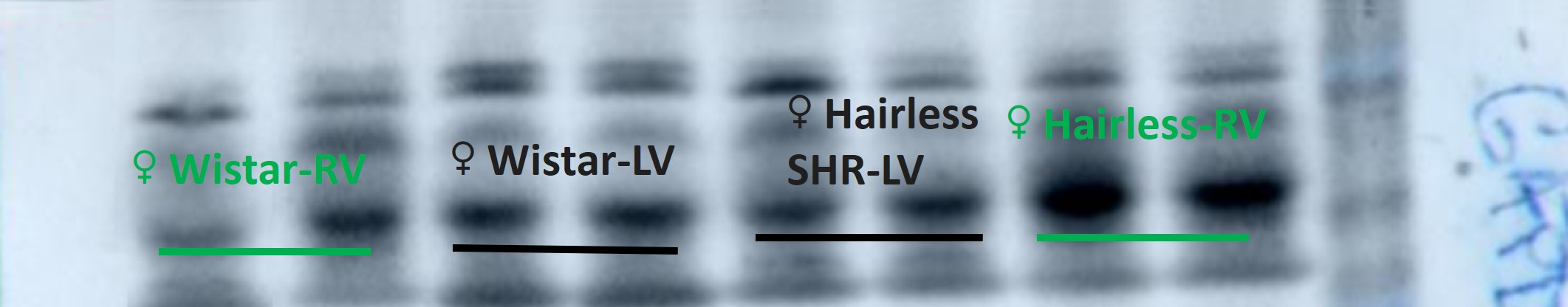

Supplement: Supplementary file 1 [file biomolecules-14-01509-s001.zip › GAPDH to tCx43 FEMALE_membrane 1.jpg]

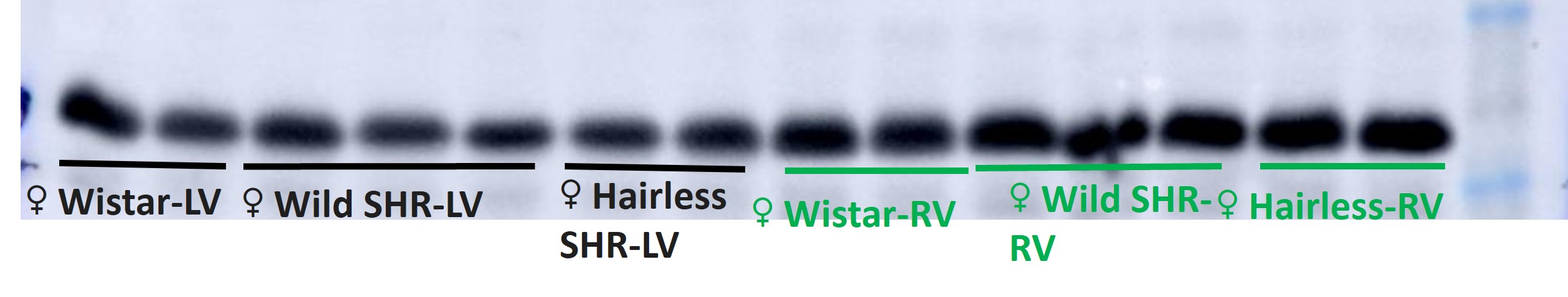

Supplement: Supplementary file 1 [file biomolecules-14-01509-s001.zip › GAPDH to tCx43 FEMALE_membrane 2.jpg]

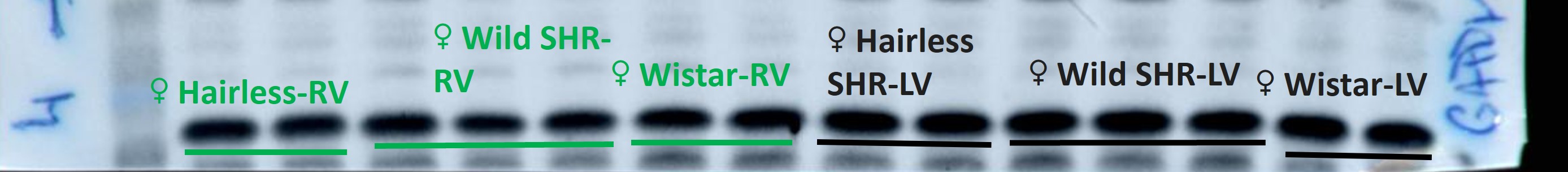

Supplement: Supplementary file 1 [file biomolecules-14-01509-s001.zip › GAPDH to tCx43 FEMALE_membrane 3.jpg]

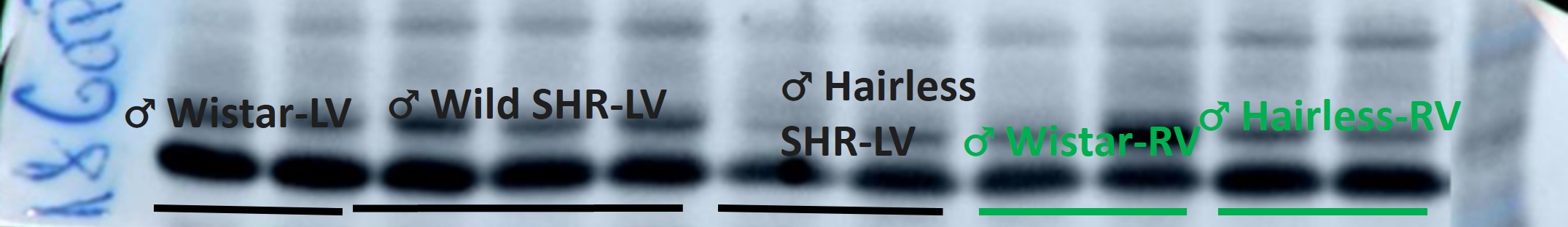

Supplement: Supplementary file 1 [file biomolecules-14-01509-s001.zip › GAPDH to tCx43 MALE_membrane 1.jpg]

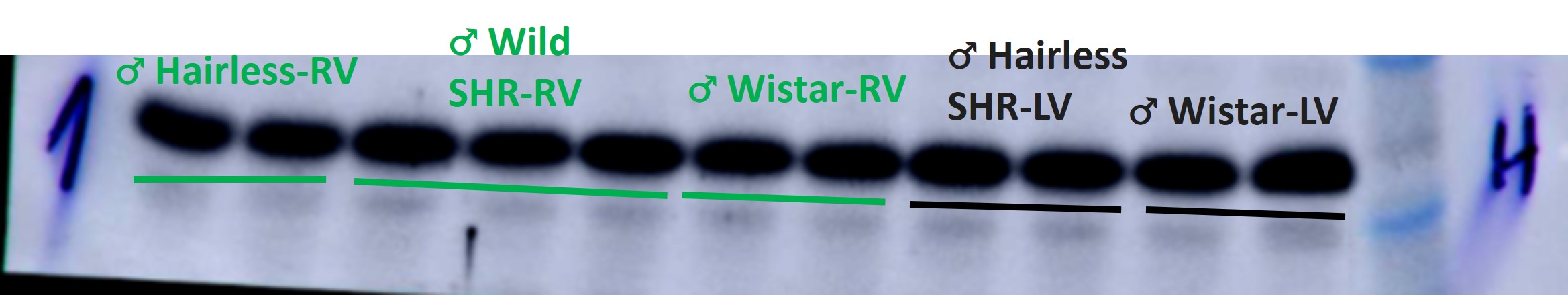

Supplement: Supplementary file 1 [file biomolecules-14-01509-s001.zip › GAPDH to tCx43 MALE_membrane 2.jpg]

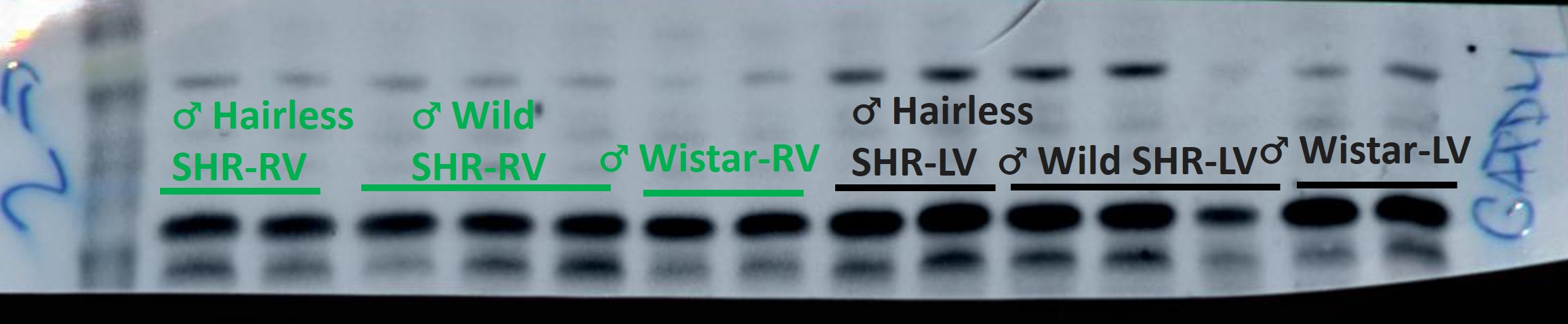

Supplement: Supplementary file 1 [file biomolecules-14-01509-s001.zip › GAPDH to tCx43 MALE_membrane 3.jpg]

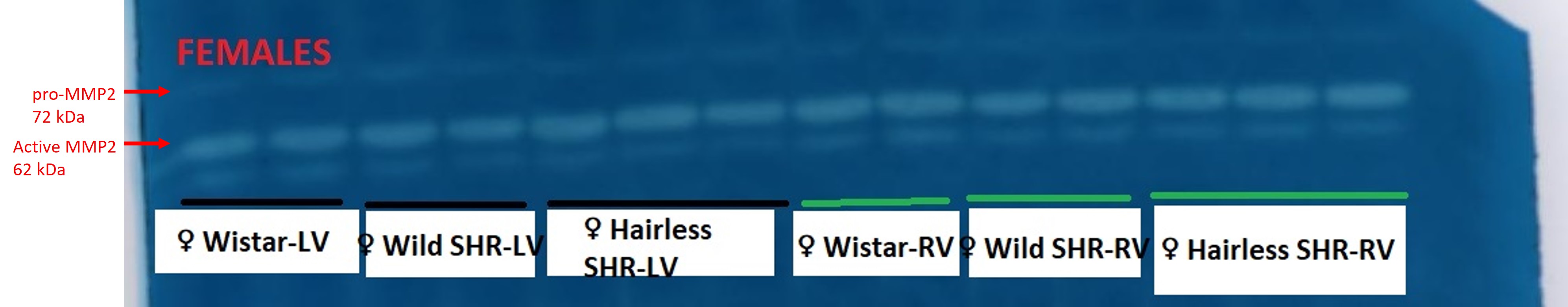

Supplement: Supplementary file 1 [file biomolecules-14-01509-s001.zip › MMP2 activity FEMALE_gel 1.jpg]

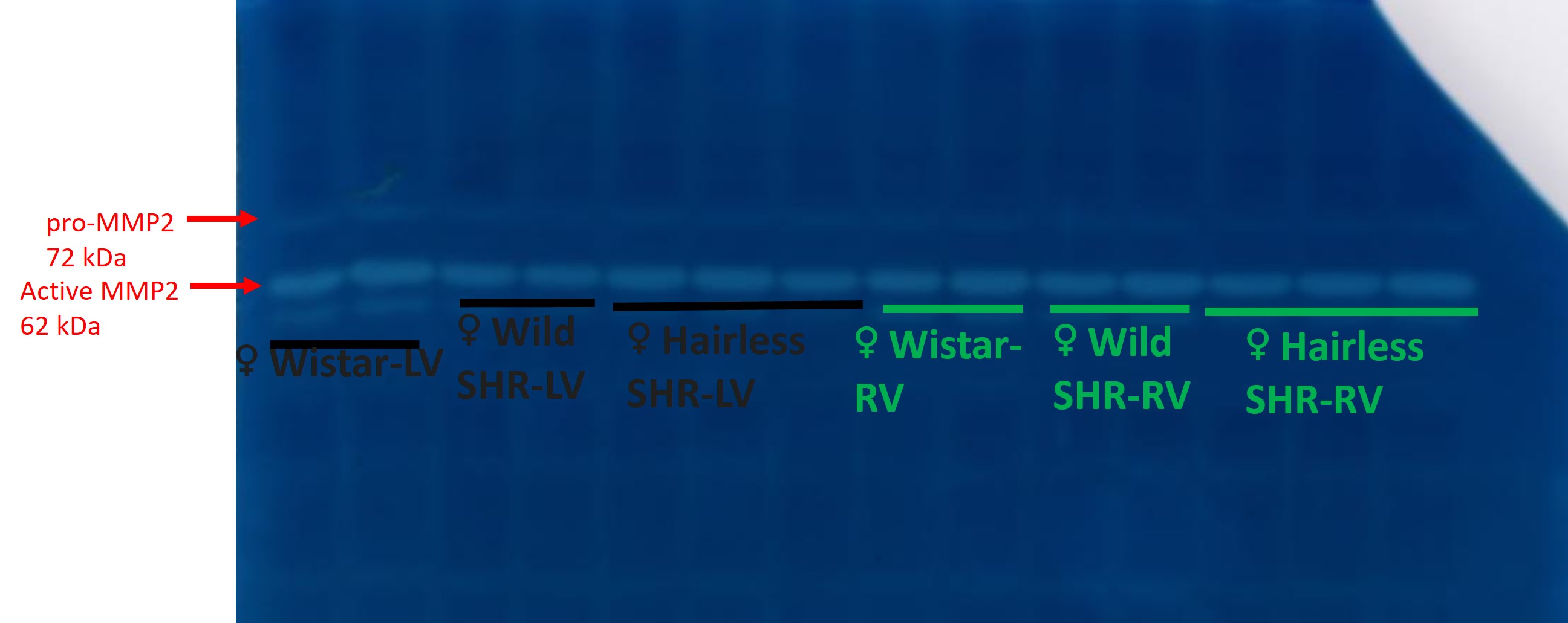

Supplement: Supplementary file 1 [file biomolecules-14-01509-s001.zip › MMP2 activity FEMALE_gel 2.jpg]

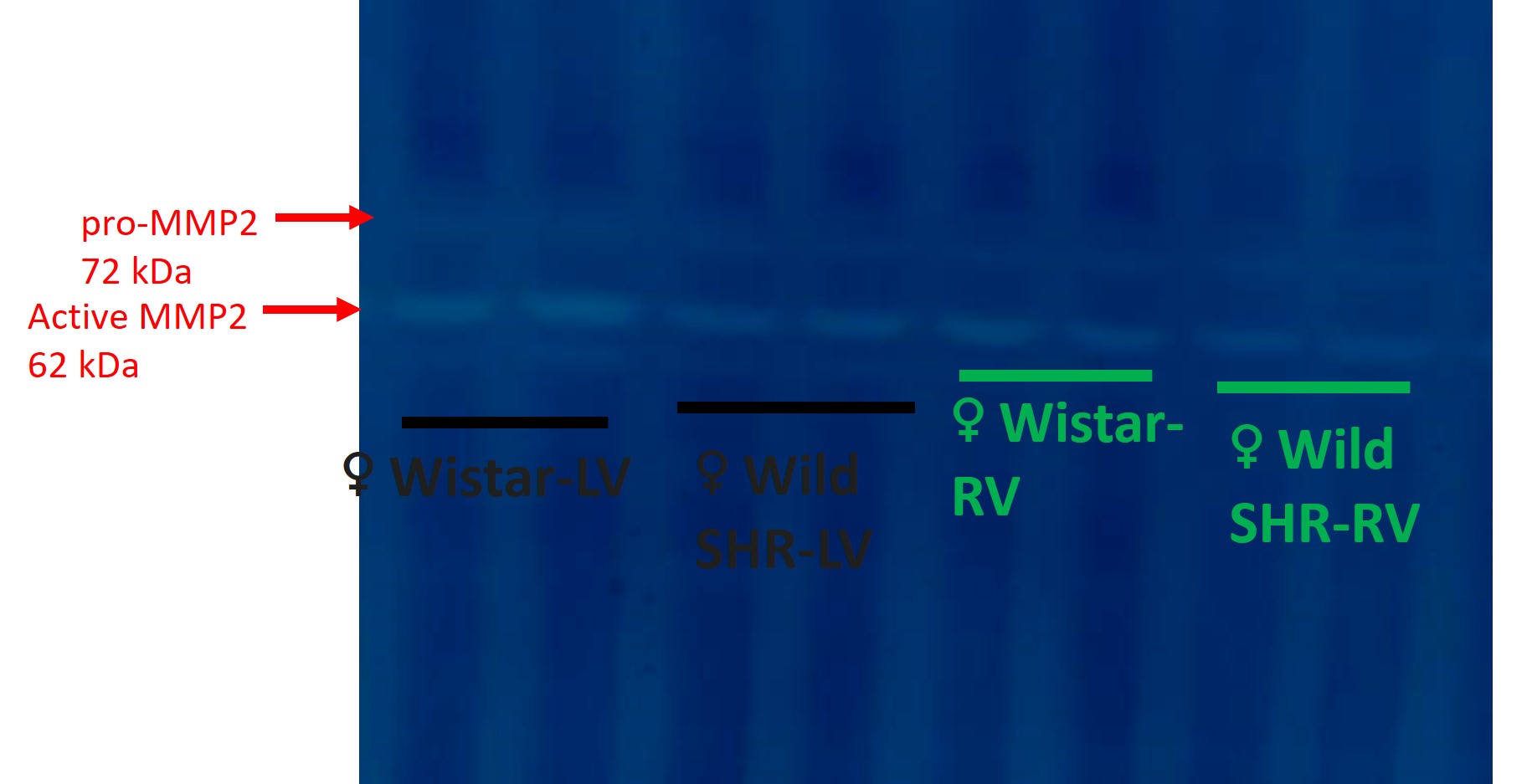

Supplement: Supplementary file 1 [file biomolecules-14-01509-s001.zip › MMP2 activity FEMALE_gel 3.jpg]

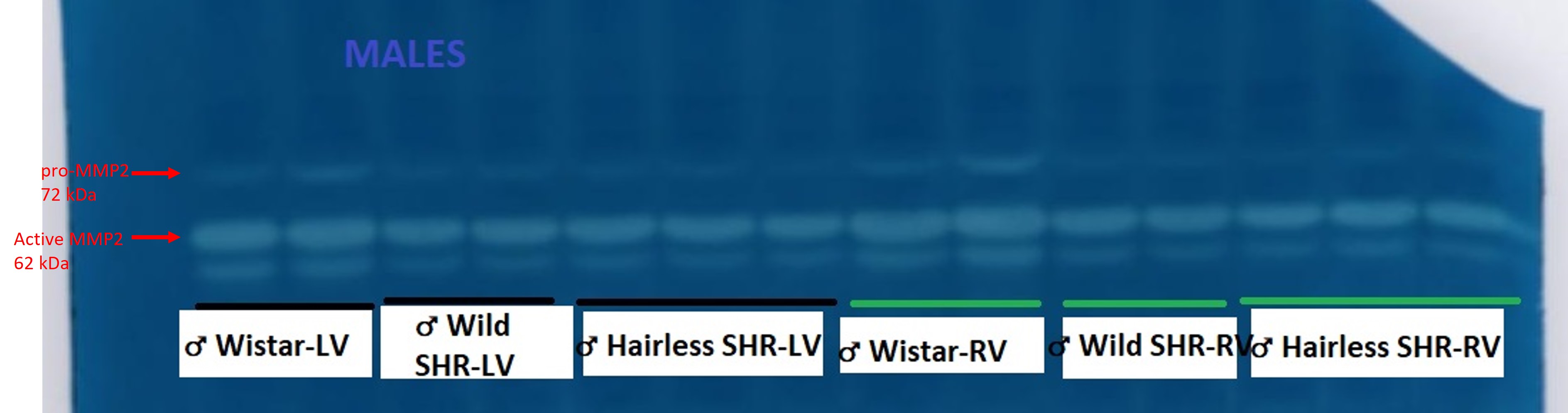

Supplement: Supplementary file 1 [file biomolecules-14-01509-s001.zip › MMP2 activity MALE_gel 1.jpg]

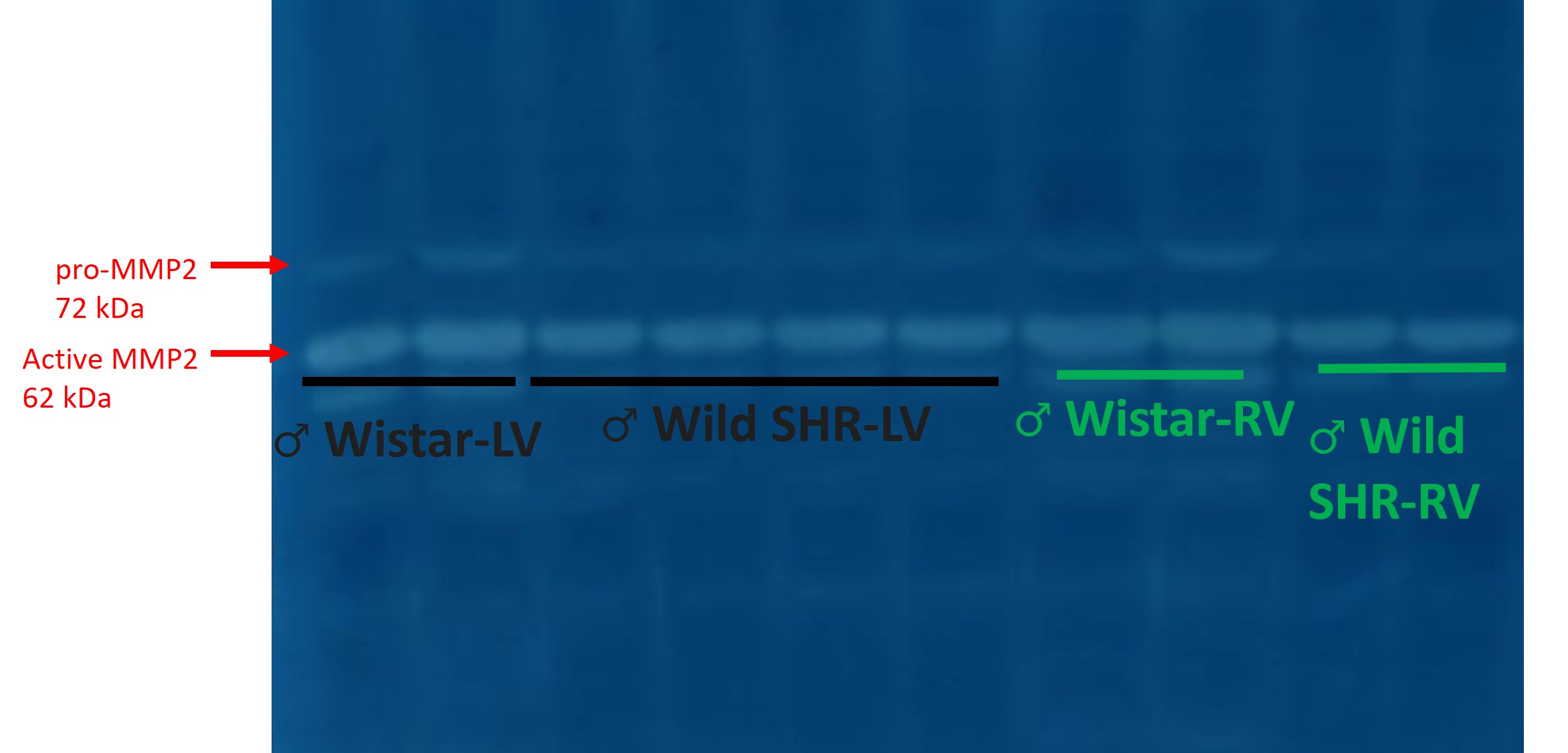

Supplement: Supplementary file 1 [file biomolecules-14-01509-s001.zip › MMP2 activity MALE_gel 2.jpg]

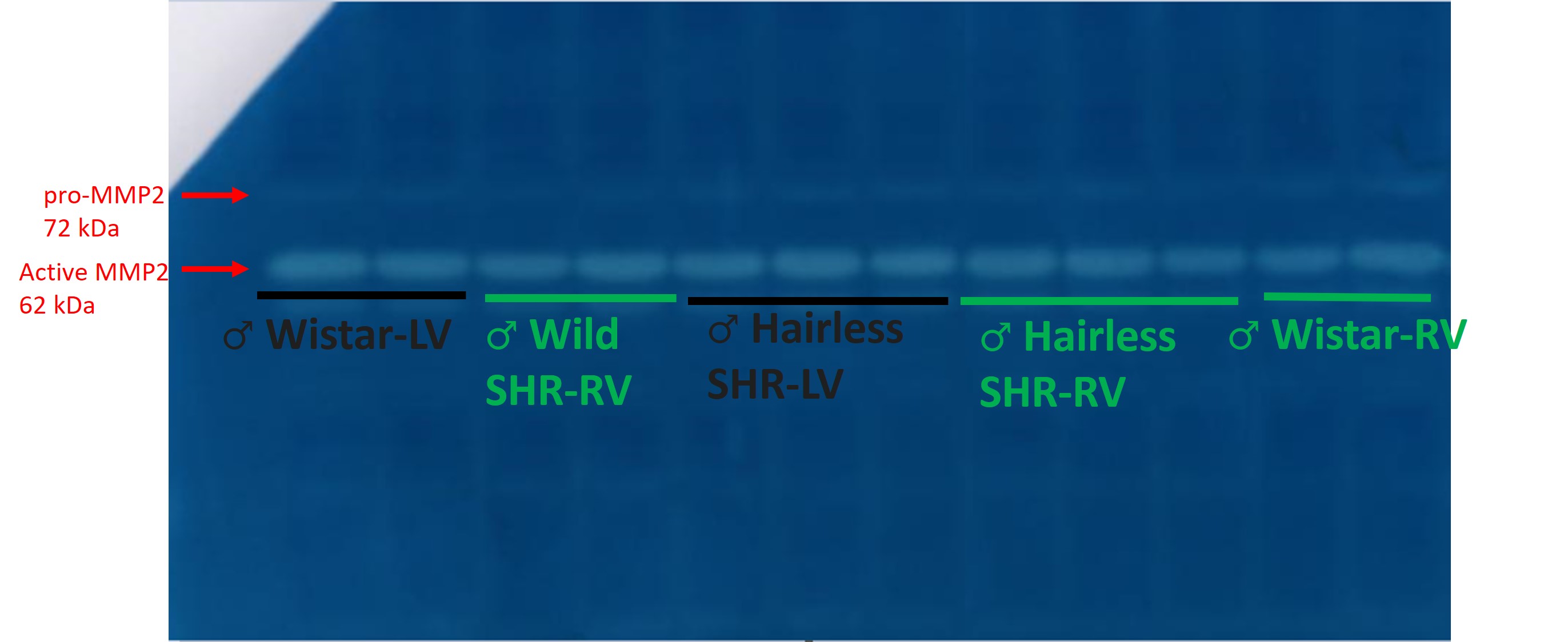

Supplement: Supplementary file 1 [file biomolecules-14-01509-s001.zip › MMP2 activity MALE_gel 3.jpg]

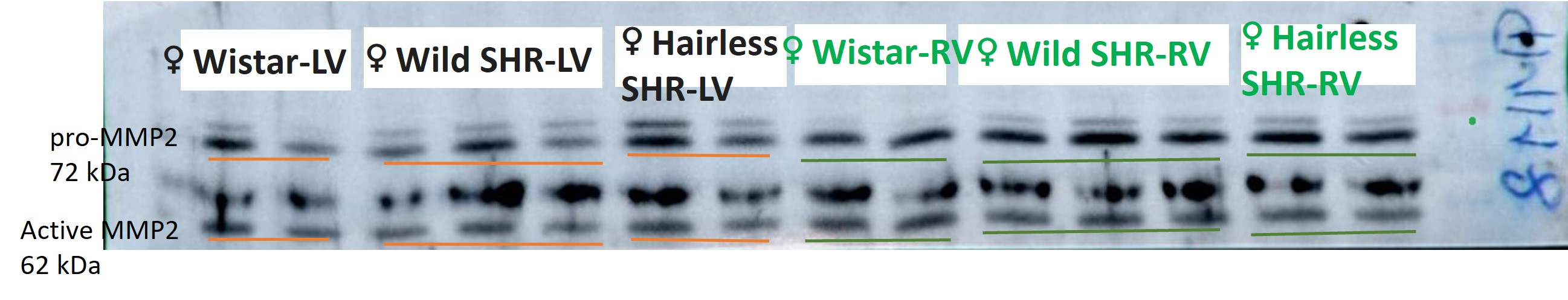

Supplement: Supplementary file 1 [file biomolecules-14-01509-s001.zip › MMP2 FEMALE_membrane 1.jpg]

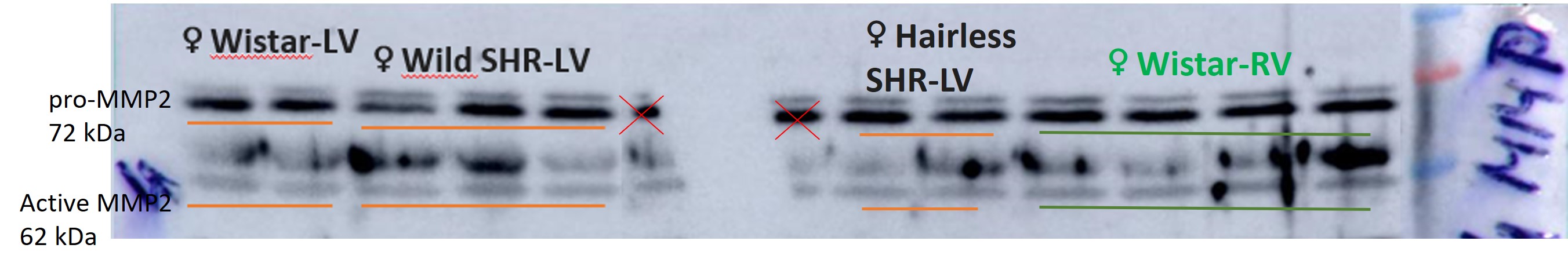

Supplement: Supplementary file 1 [file biomolecules-14-01509-s001.zip › MMP2 FEMALE_membrane 2.jpg]

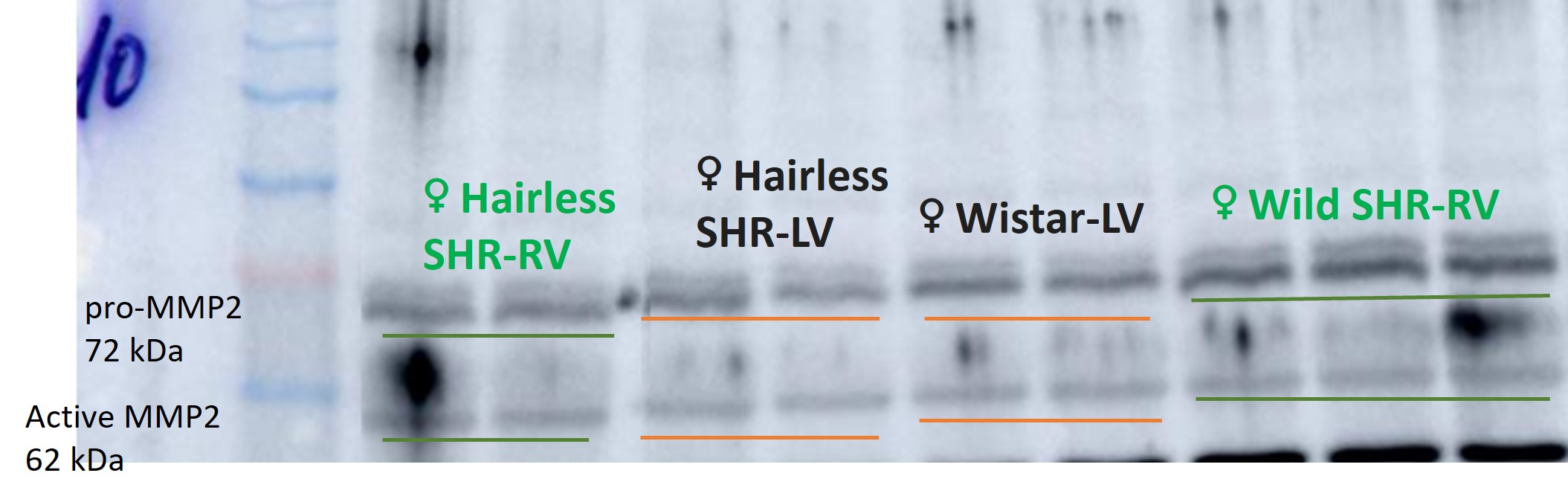

Supplement: Supplementary file 1 [file biomolecules-14-01509-s001.zip › MMP2 FEMALE_membrane 3.jpg]

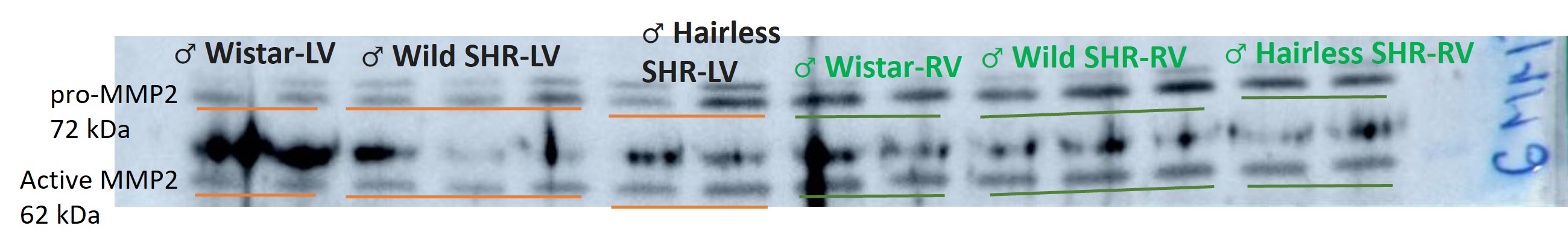

Supplement: Supplementary file 1 [file biomolecules-14-01509-s001.zip › MMP2 MALE_membrane 1.jpg]

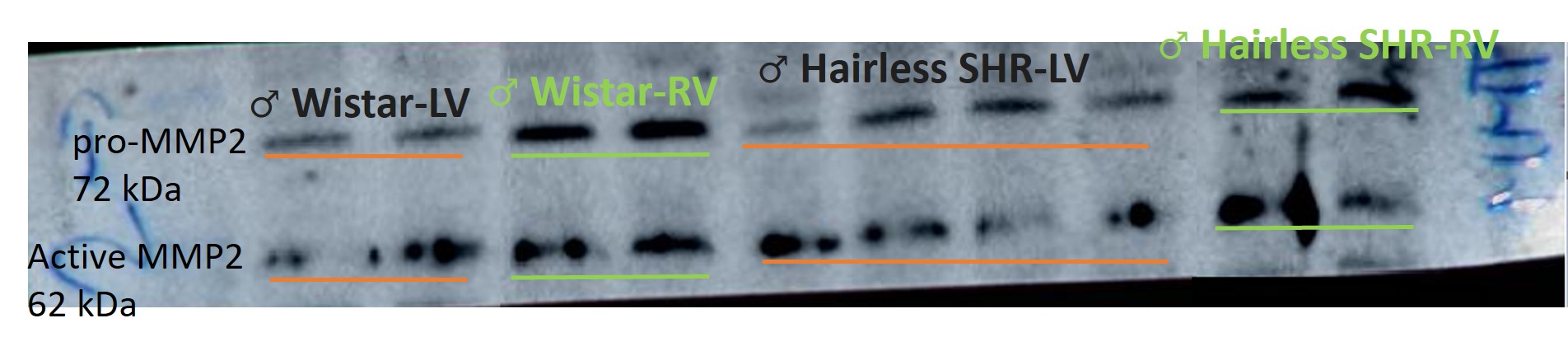

Supplement: Supplementary file 1 [file biomolecules-14-01509-s001.zip › MMP2 MALE_membrane 2.jpg]

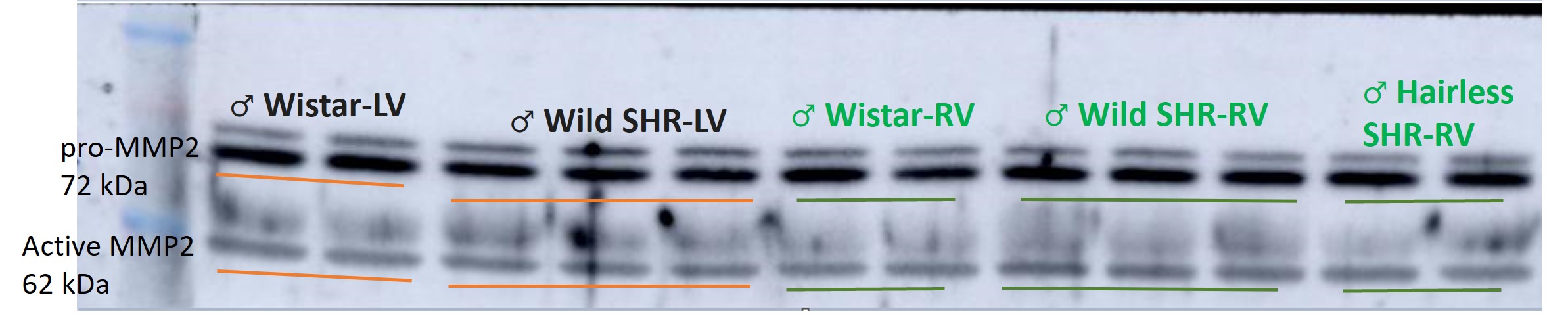

Supplement: Supplementary file 1 [file biomolecules-14-01509-s001.zip › MMP2 MALE_membrane 3.jpg]

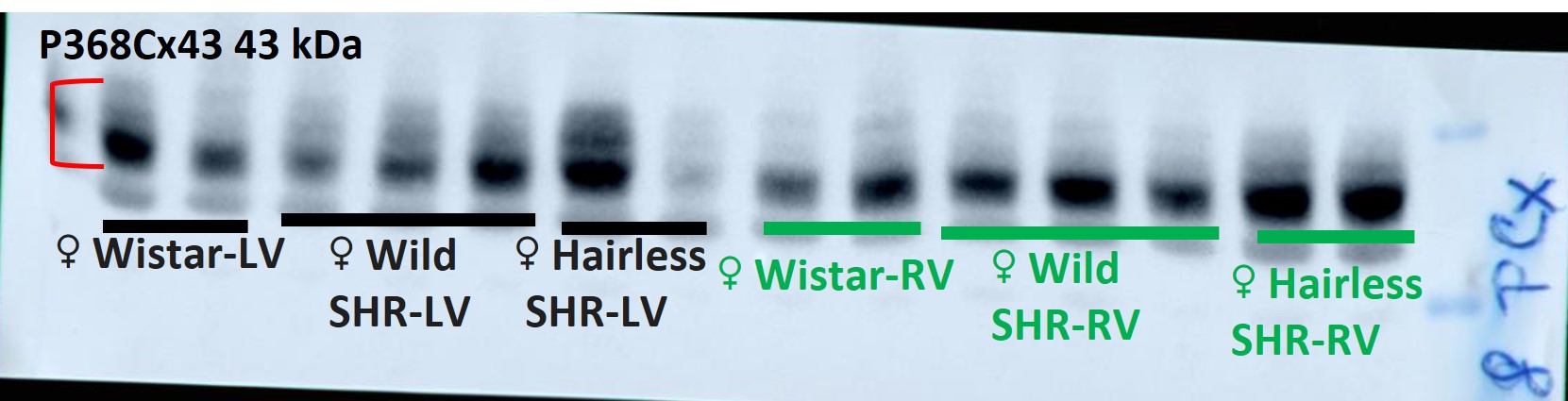

Supplement: Supplementary file 1 [file biomolecules-14-01509-s001.zip › p368Cx43 FEMALE_membrane 1.jpg]

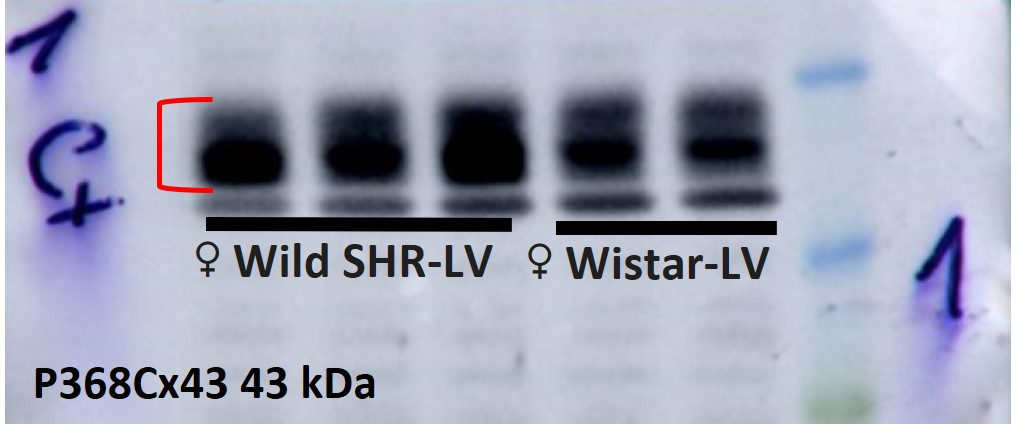

Supplement: Supplementary file 1 [file biomolecules-14-01509-s001.zip › p368Cx43 FEMALE_membrane 2.jpg]

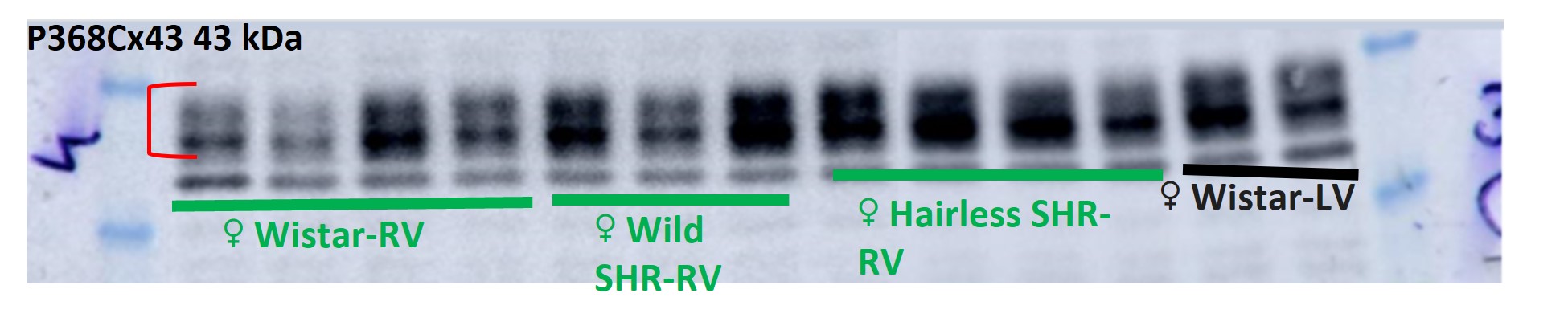

Supplement: Supplementary file 1 [file biomolecules-14-01509-s001.zip › p368Cx43 FEMALE_membrane 3.jpg]

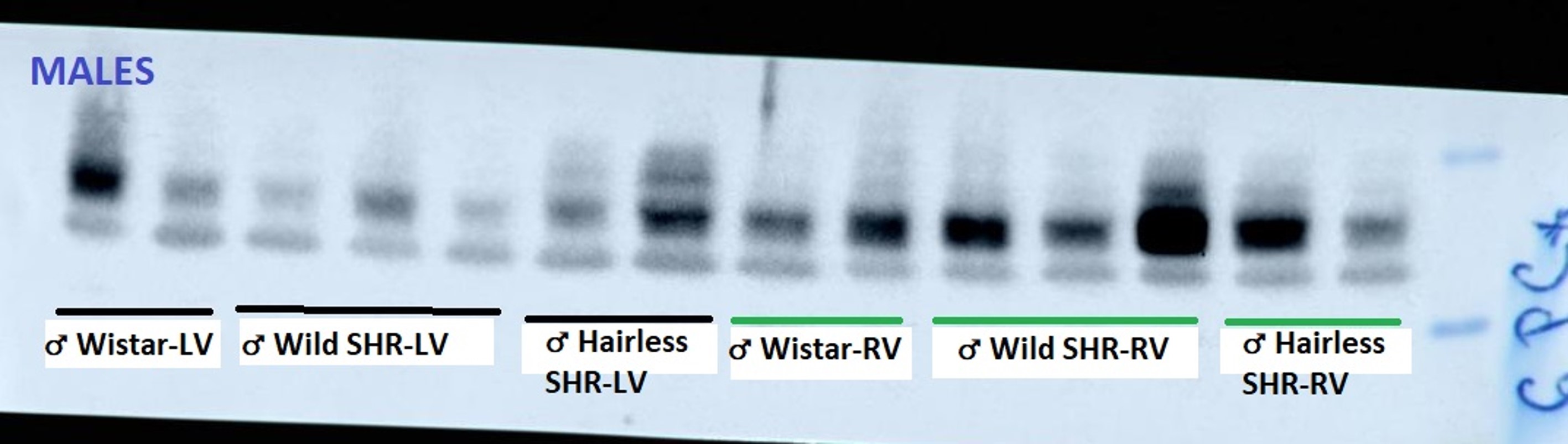

Supplement: Supplementary file 1 [file biomolecules-14-01509-s001.zip › p368Cx43 MALE_membrane 1.jpg]

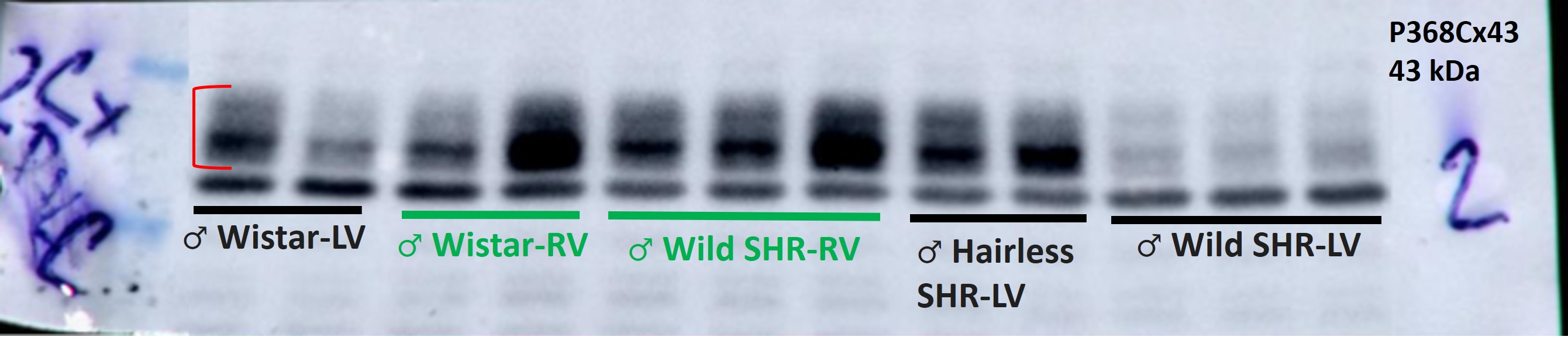

Supplement: Supplementary file 1 [file biomolecules-14-01509-s001.zip › p368Cx43 MALE_membrane 2.jpg]

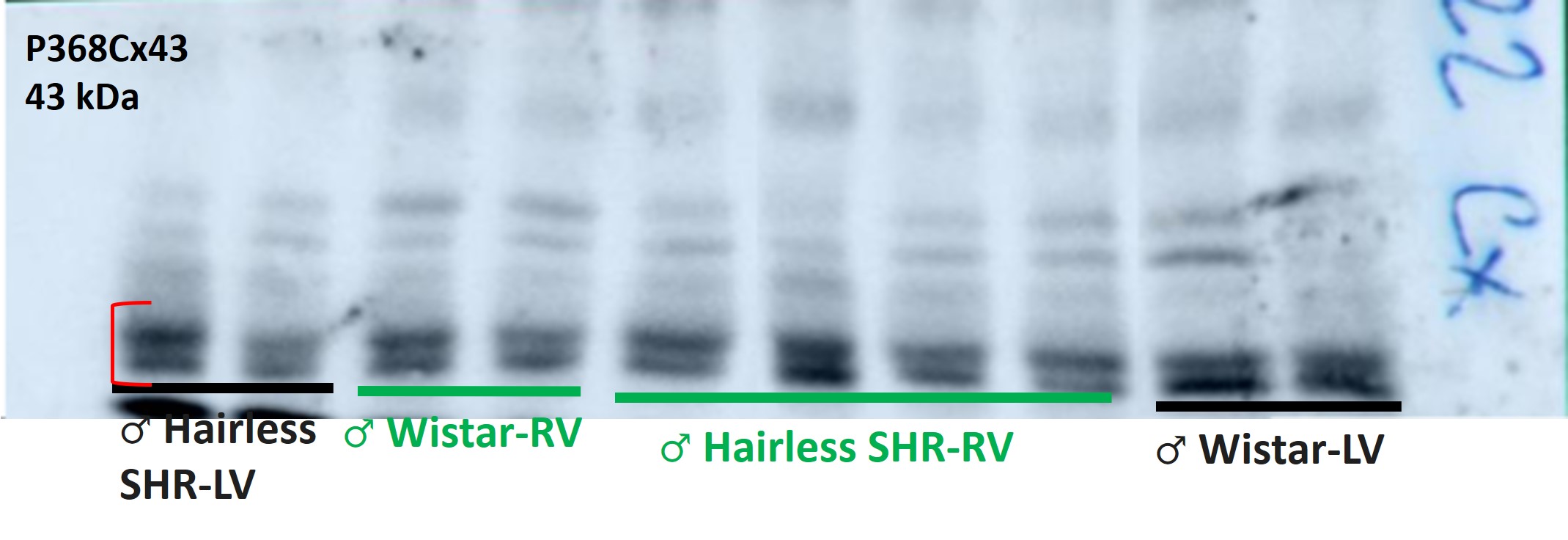

Supplement: Supplementary file 1 [file biomolecules-14-01509-s001.zip › p368Cx43 MALE_membrane 3.jpg]

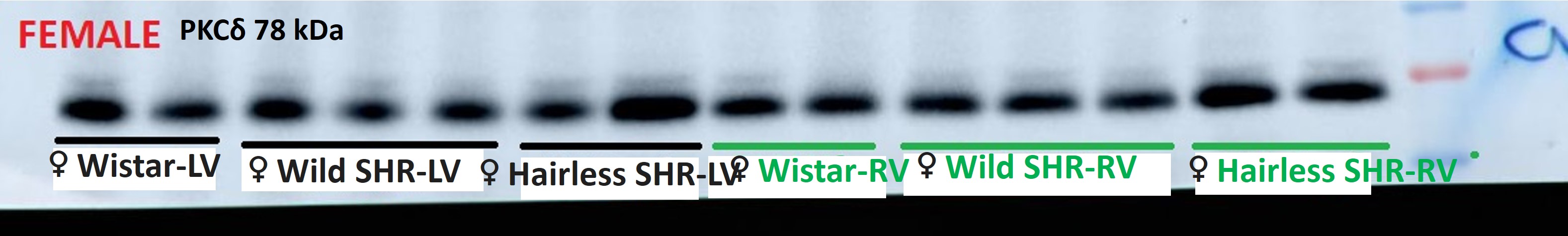

Supplement: Supplementary file 1 [file biomolecules-14-01509-s001.zip › PKCdelta FEMALE_mambrane 1.jpg]

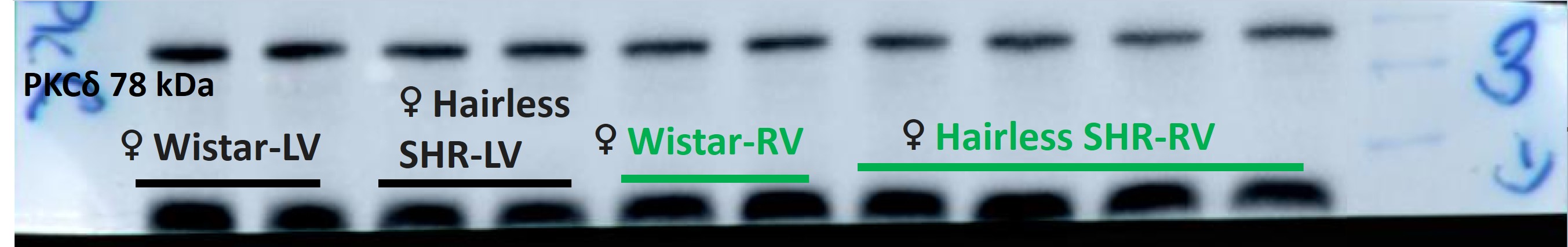

Supplement: Supplementary file 1 [file biomolecules-14-01509-s001.zip › PKCdelta FEMALE_mambrane 2.jpg]

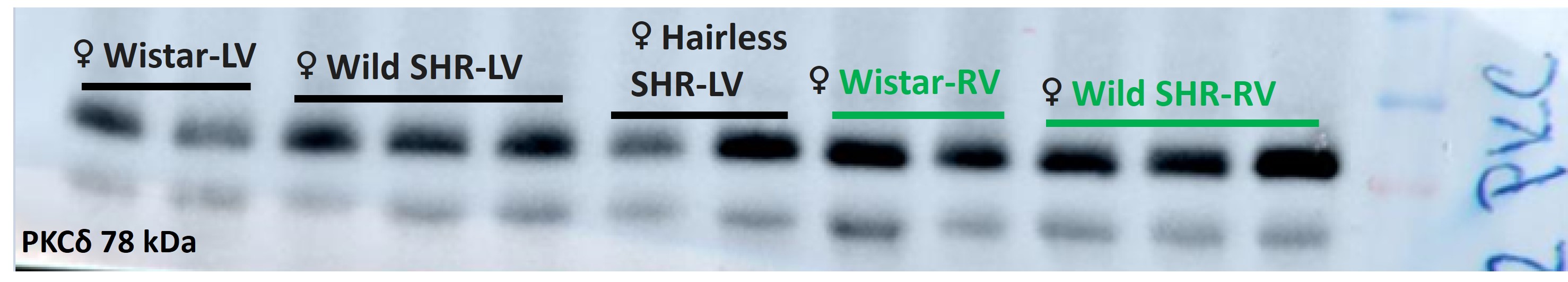

Supplement: Supplementary file 1 [file biomolecules-14-01509-s001.zip › PKCdelta FEMALE_mambrane 3.jpg]

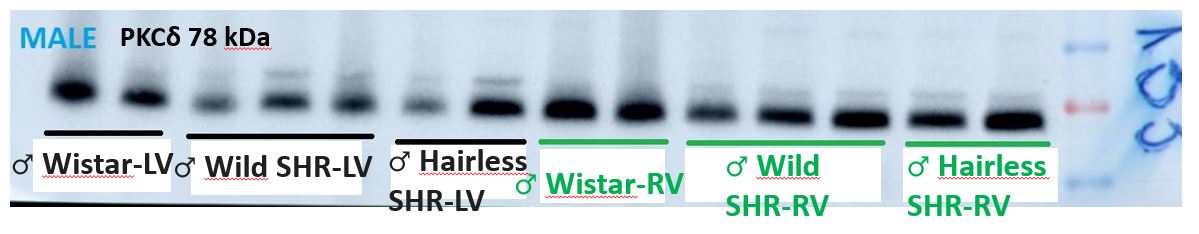

Supplement: Supplementary file 1 [file biomolecules-14-01509-s001.zip › PKCdelta MALE_mambrane 1.JPG]

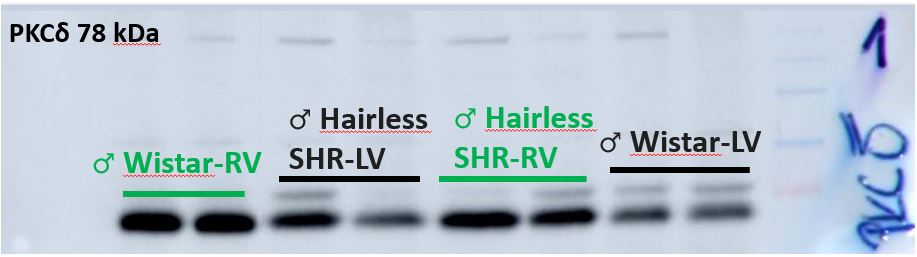

Supplement: Supplementary file 1 [file biomolecules-14-01509-s001.zip › PKCdelta MALE_mambrane 2.JPG]

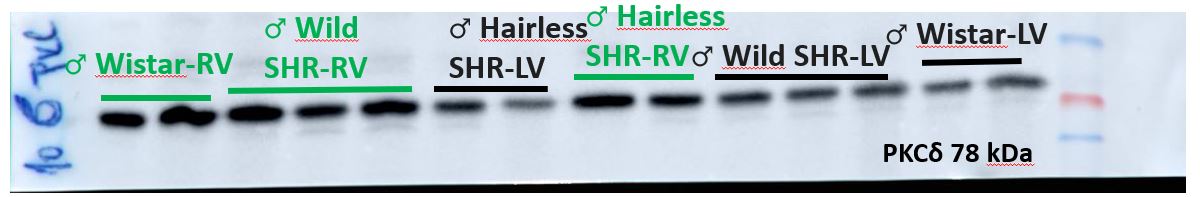

Supplement: Supplementary file 1 [file biomolecules-14-01509-s001.zip › PKCdelta MALE_mambrane 3.JPG]

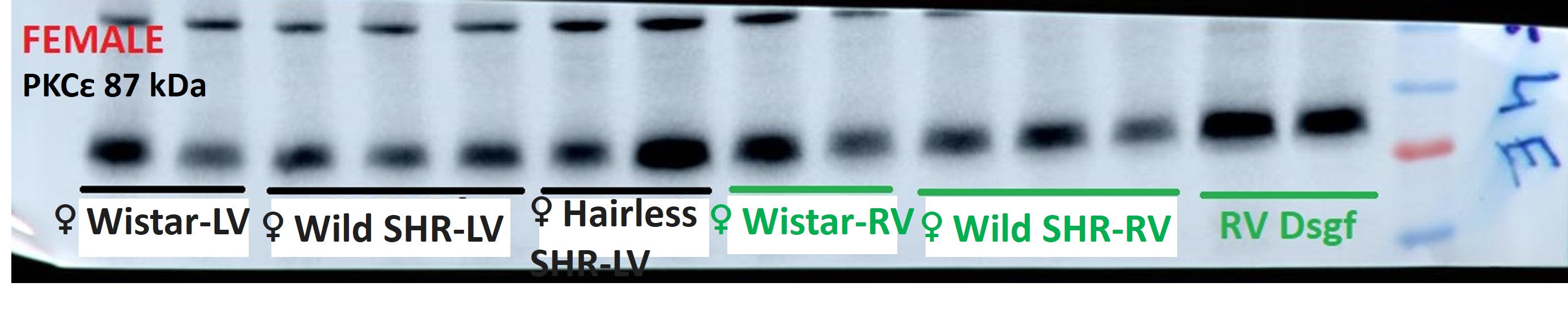

Supplement: Supplementary file 1 [file biomolecules-14-01509-s001.zip › PKCepsilon FEMALE_mambrane 1.jpg]

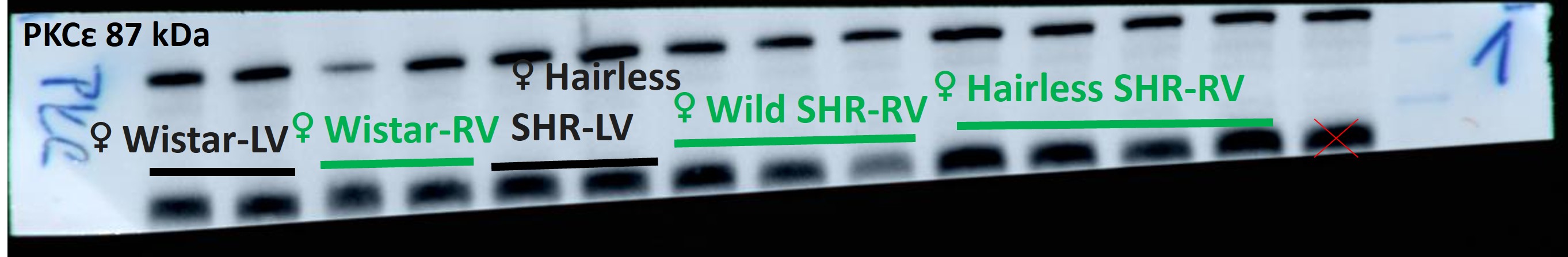

Supplement: Supplementary file 1 [file biomolecules-14-01509-s001.zip › PKCepsilon FEMALE_mambrane 2.jpg]

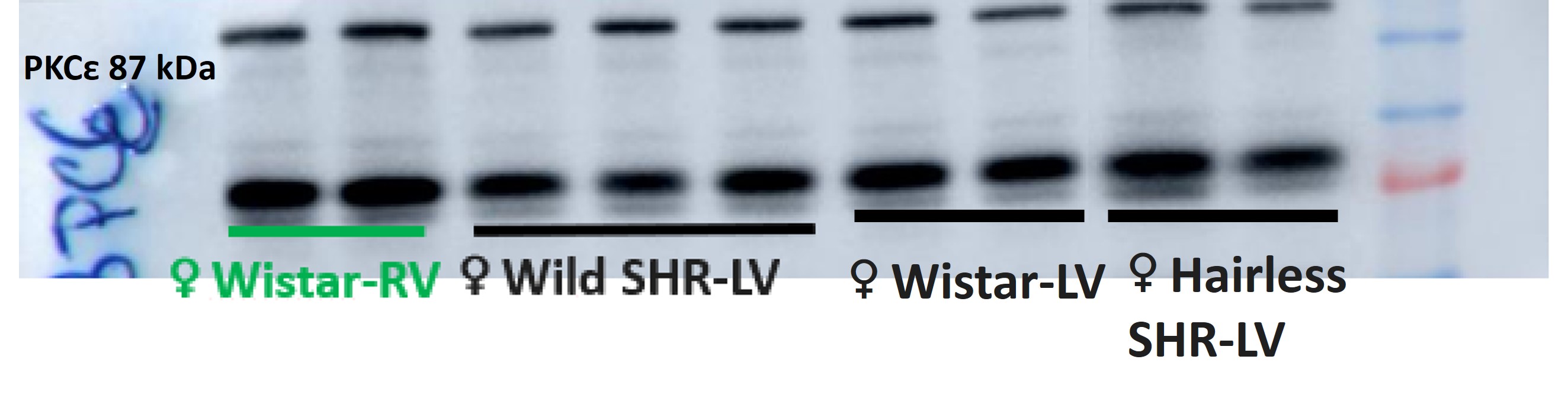

Supplement: Supplementary file 1 [file biomolecules-14-01509-s001.zip › PKCepsilon FEMALE_mambrane 3.jpg]

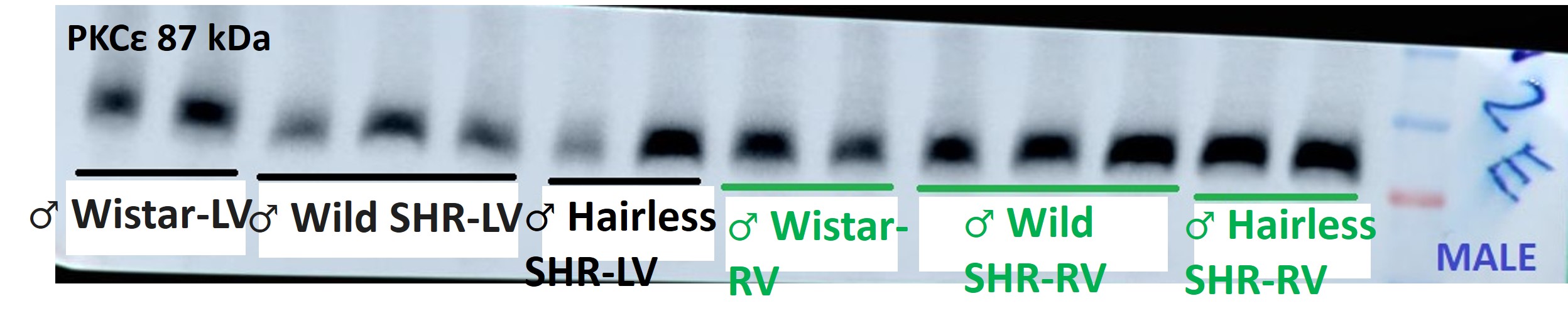

Supplement: Supplementary file 1 [file biomolecules-14-01509-s001.zip › PKCepsilon MALE_mambrane 1.jpg]

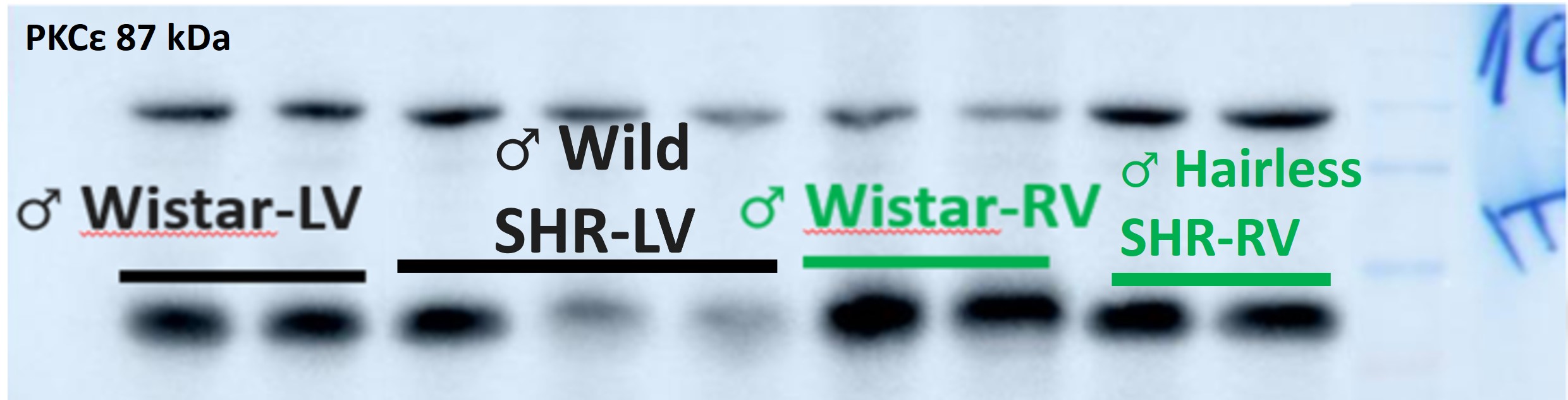

Supplement: Supplementary file 1 [file biomolecules-14-01509-s001.zip › PKCepsilon MALE_mambrane 2.jpg]

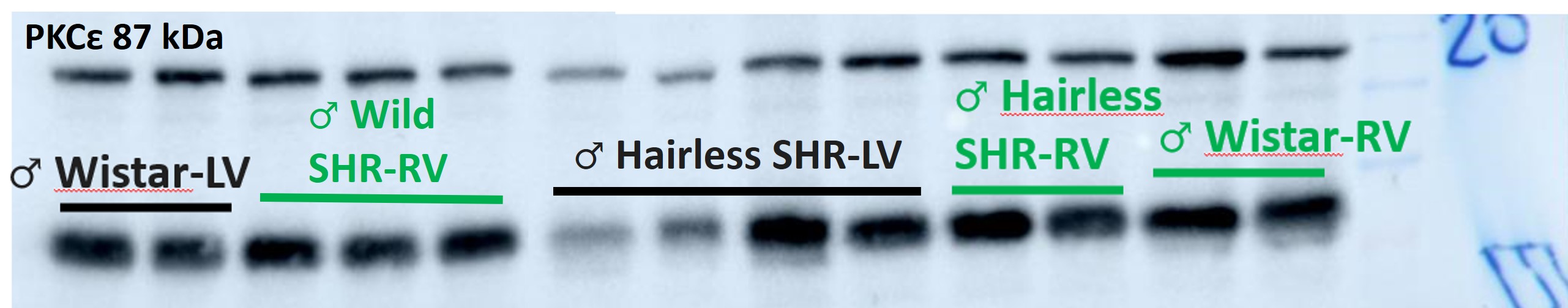

Supplement: Supplementary file 1 [file biomolecules-14-01509-s001.zip › PKCepsilon MALE_mambrane 3.jpg]

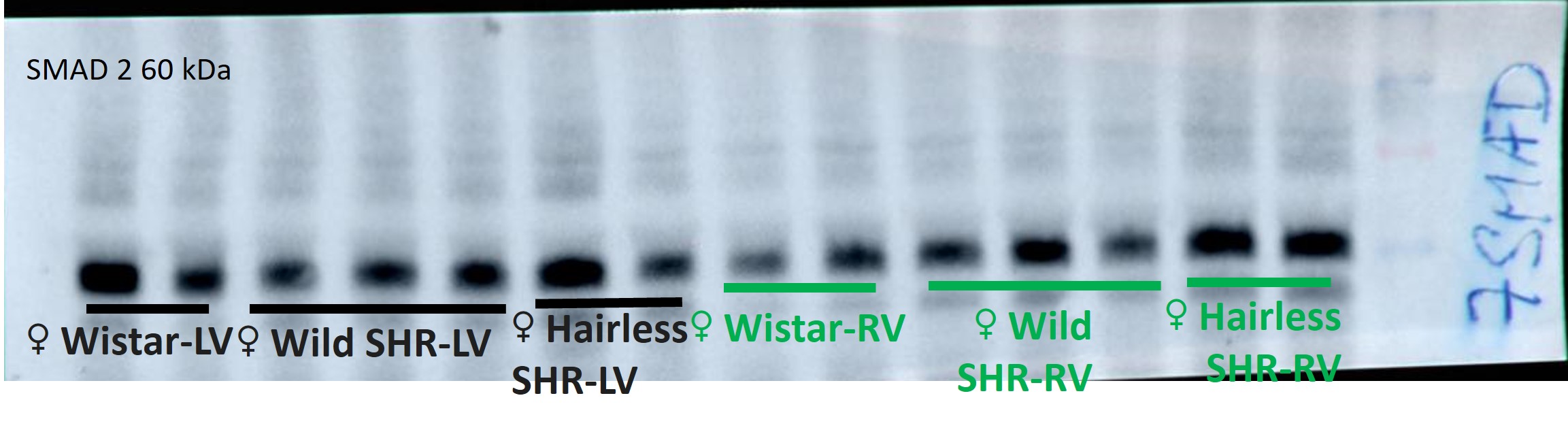

Supplement: Supplementary file 1 [file biomolecules-14-01509-s001.zip › SMAD 2 FEMALE_membrane 1.jpg]

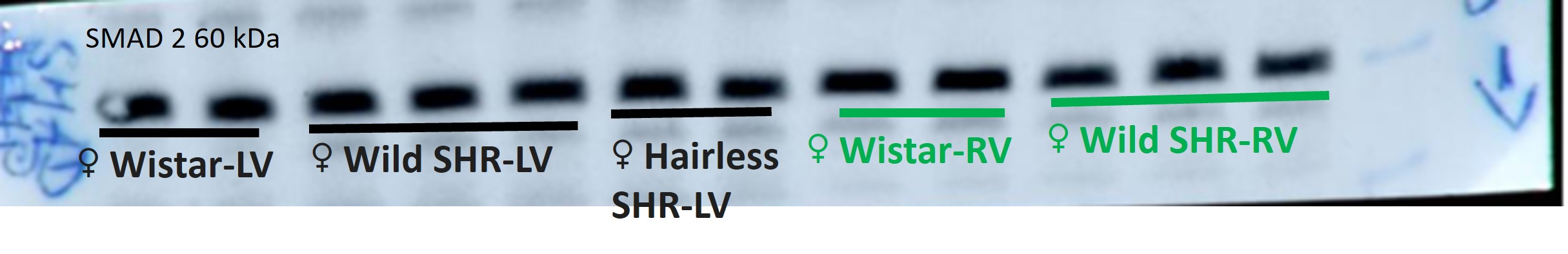

Supplement: Supplementary file 1 [file biomolecules-14-01509-s001.zip › SMAD 2 FEMALE_membrane 2.jpg]

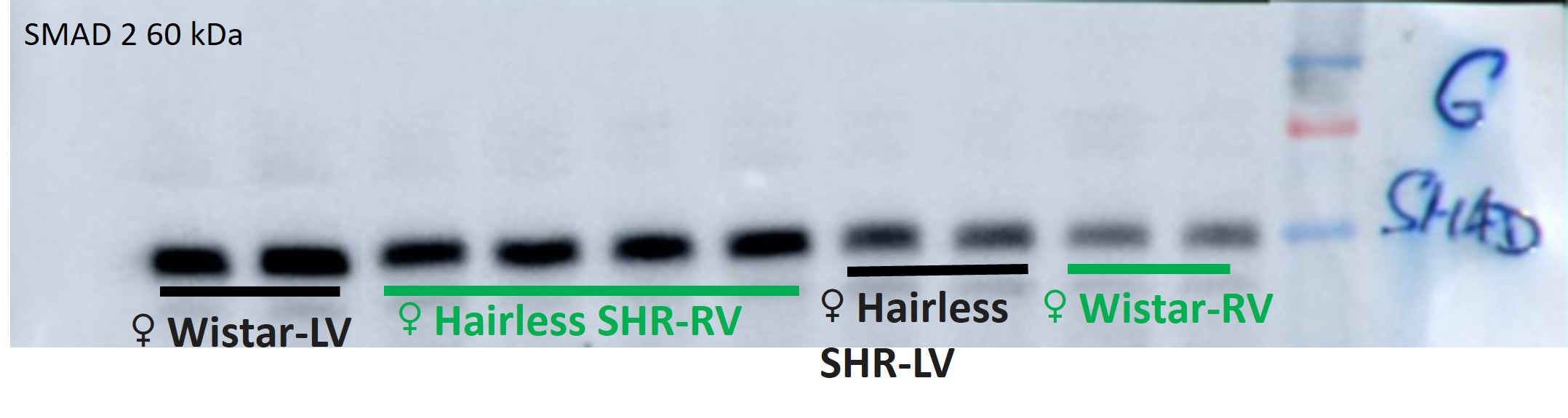

Supplement: Supplementary file 1 [file biomolecules-14-01509-s001.zip › SMAD 2 FEMALE_membrane 3.jpg]

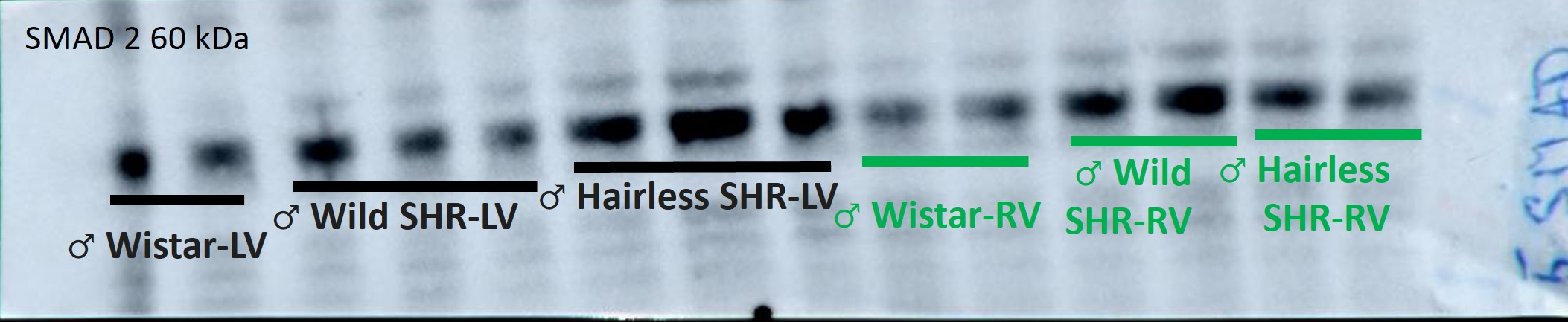

Supplement: Supplementary file 1 [file biomolecules-14-01509-s001.zip › SMAD 2 MALE_membrane 1.jpg]

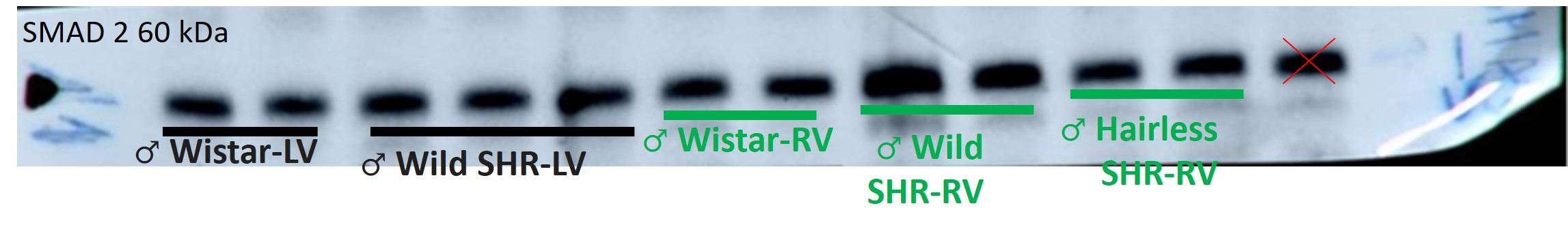

Supplement: Supplementary file 1 [file biomolecules-14-01509-s001.zip › SMAD 2 MALE_membrane 2.jpg]

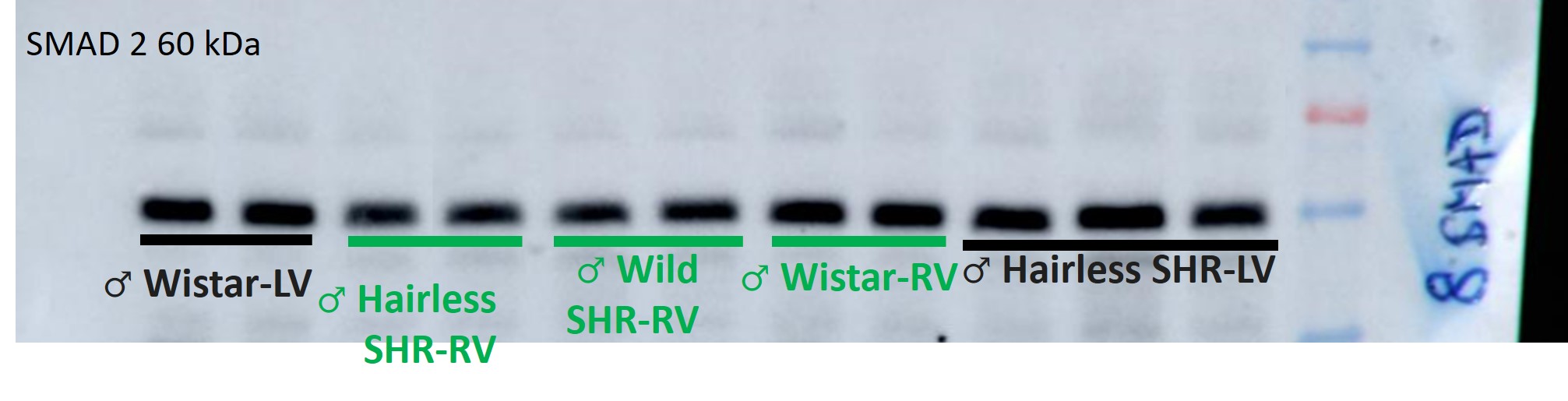

Supplement: Supplementary file 1 [file biomolecules-14-01509-s001.zip › SMAD 2 MALE_membrane 3.jpg]

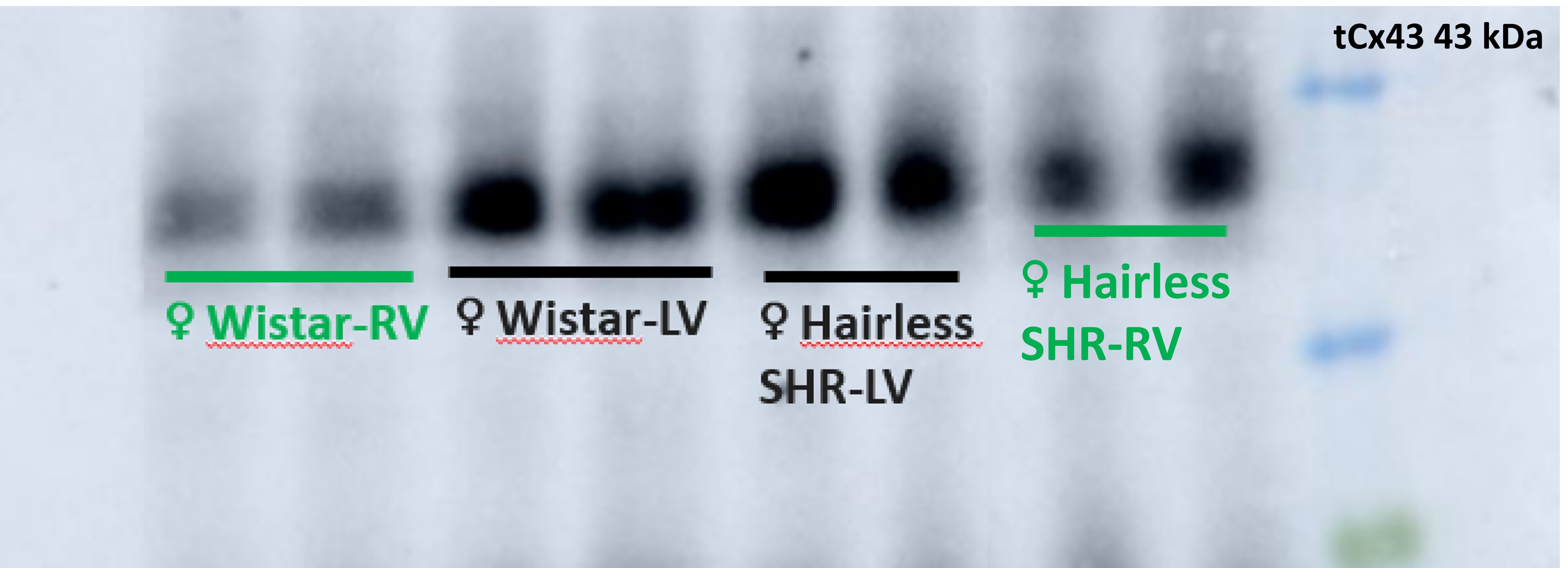

Supplement: Supplementary file 1 [file biomolecules-14-01509-s001.zip › tCx FEMALE_membrane 1.jpg]

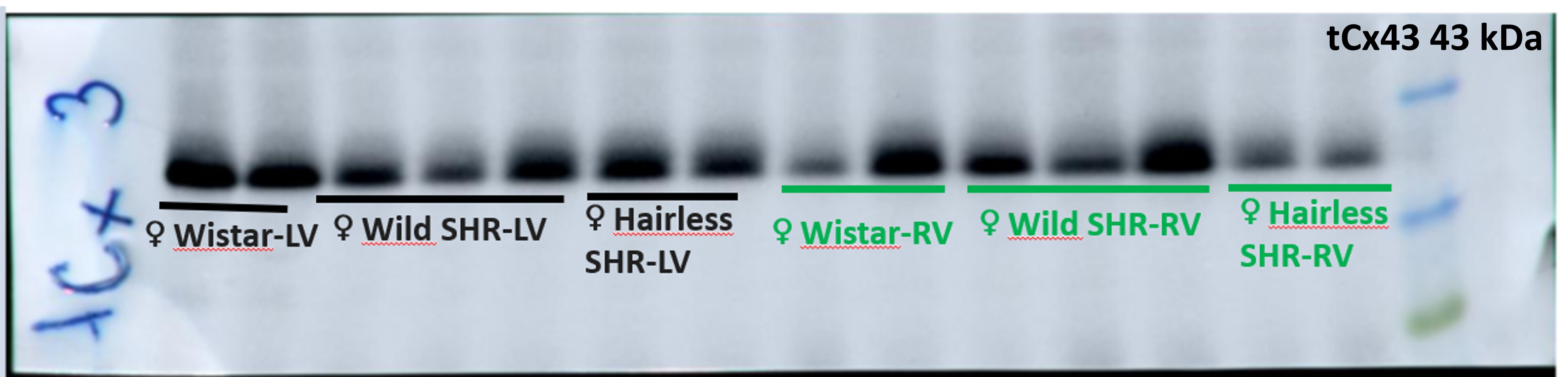

Supplement: Supplementary file 1 [file biomolecules-14-01509-s001.zip › tCx FEMALE_membrane 2.jpg]

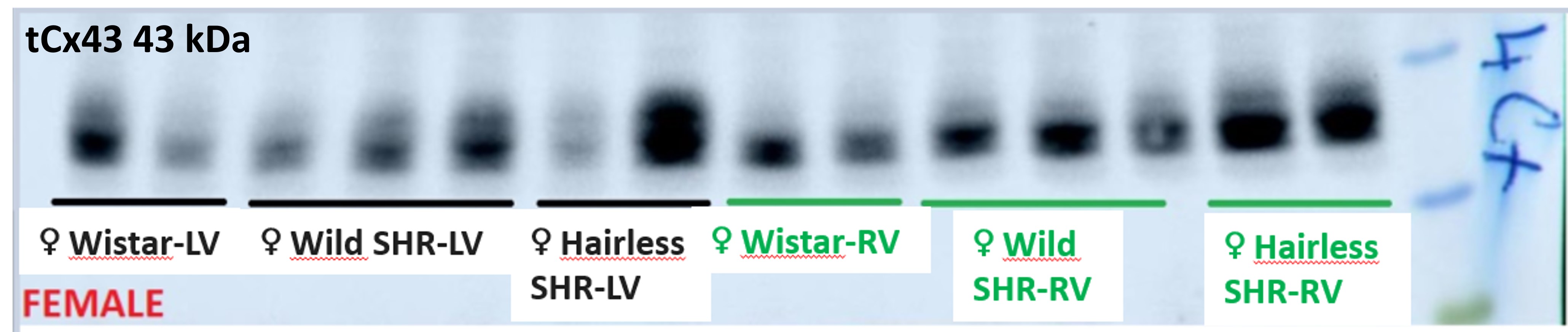

Supplement: Supplementary file 1 [file biomolecules-14-01509-s001.zip › tCx FEMALE_membrane 3.jpg]

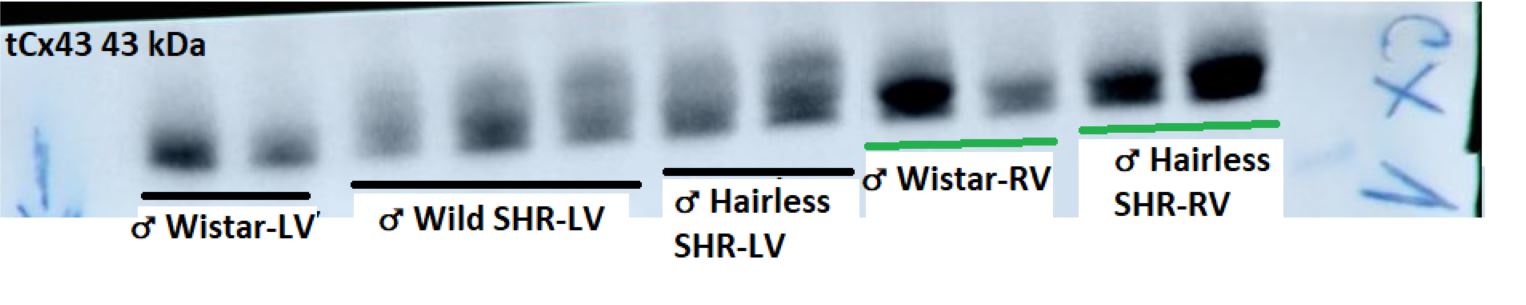

Supplement: Supplementary file 1 [file biomolecules-14-01509-s001.zip › tCx MALE_membrane 1.JPG]

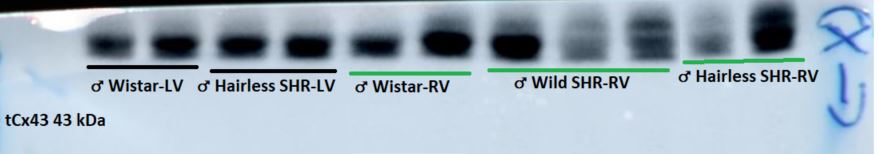

Supplement: Supplementary file 1 [file biomolecules-14-01509-s001.zip › tCx MALE_membrane 2.JPG]

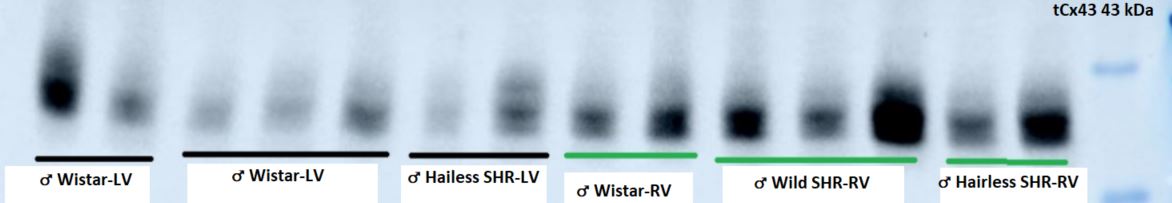

Supplement: Supplementary file 1 [file biomolecules-14-01509-s001.zip › tCx MALE_membrane 3.JPG]

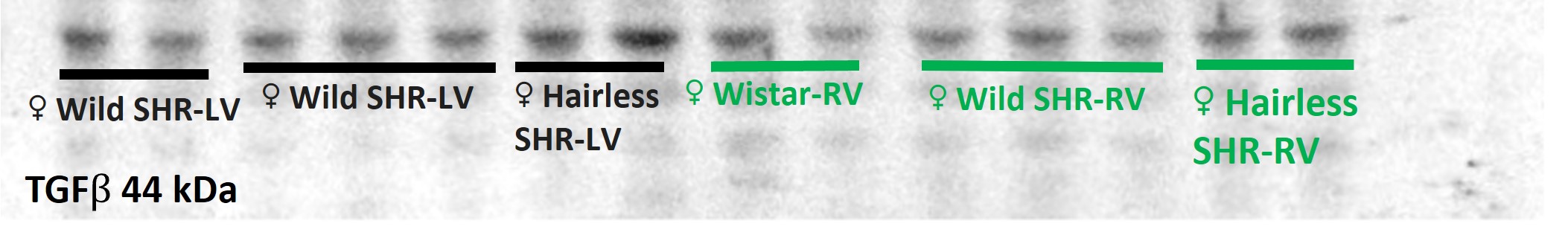

Supplement: Supplementary file 1 [file biomolecules-14-01509-s001.zip › TGFbeta FEMALE_membrane 1.jpg]

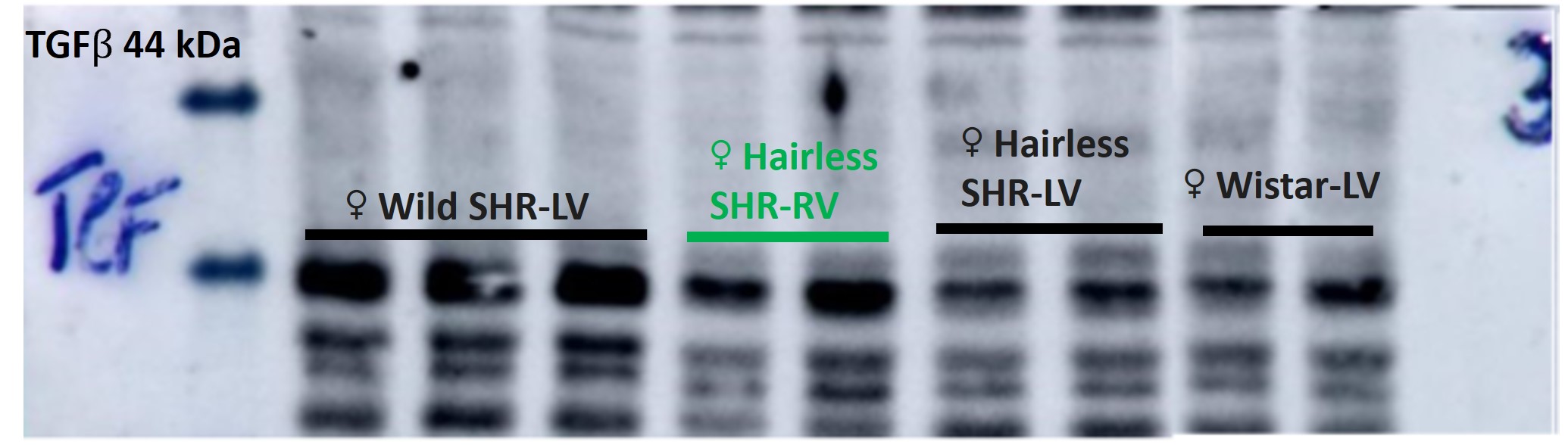

Supplement: Supplementary file 1 [file biomolecules-14-01509-s001.zip › TGFbeta FEMALE_membrane 2.jpg]

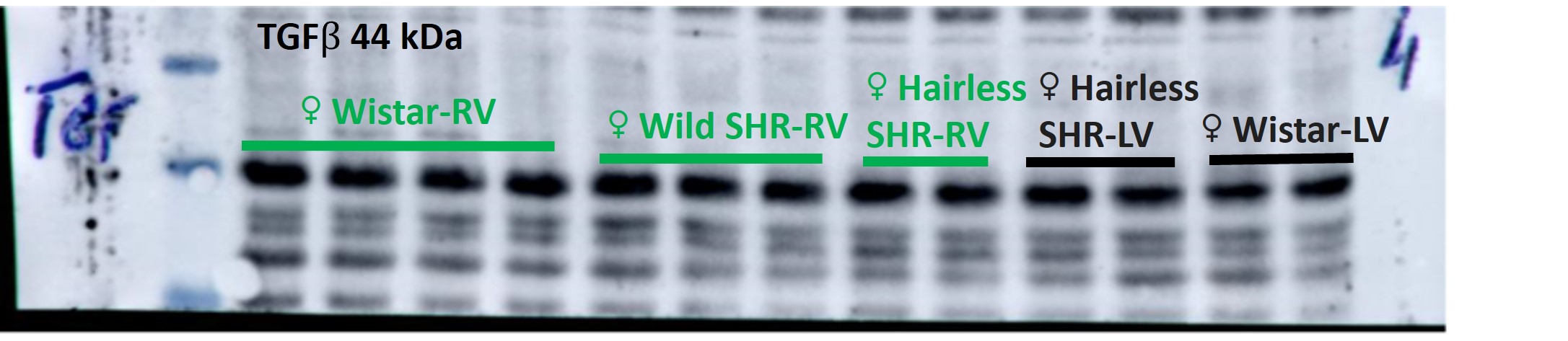

Supplement: Supplementary file 1 [file biomolecules-14-01509-s001.zip › TGFbeta FEMALE_membrane 3.jpg]

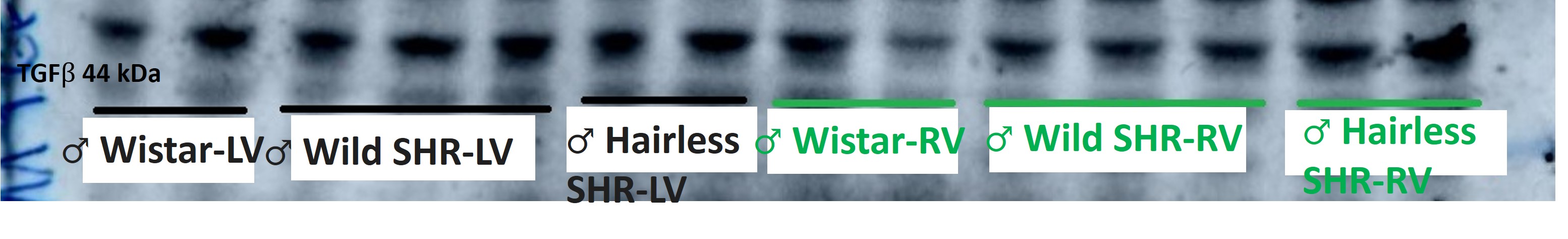

Supplement: Supplementary file 1 [file biomolecules-14-01509-s001.zip › TGFbeta MALE_membrane 1.jpg]

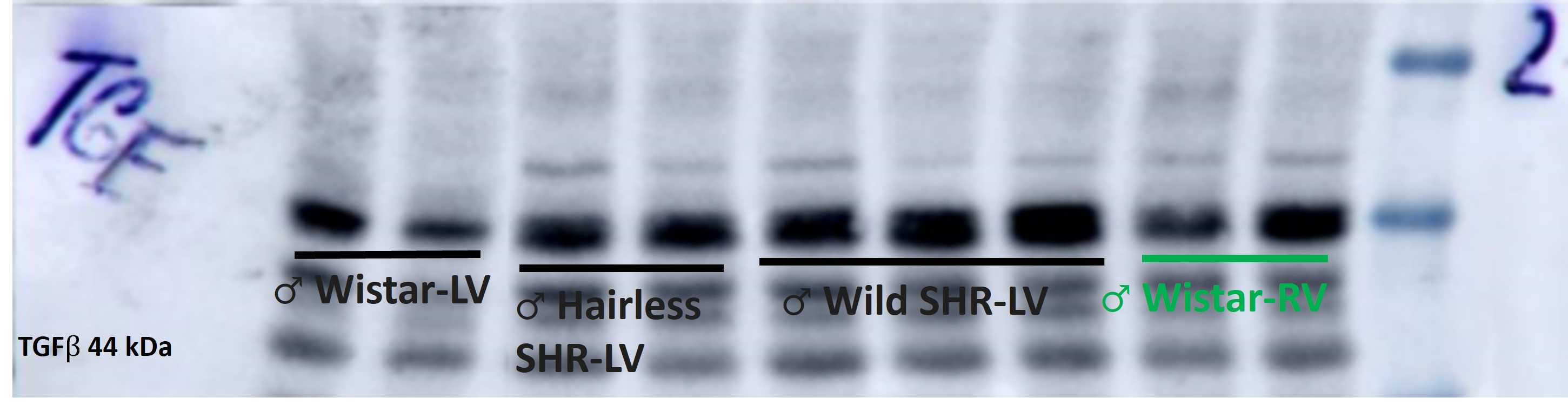

Supplement: Supplementary file 1 [file biomolecules-14-01509-s001.zip › TGFbeta MALE_membrane 2.jpg]

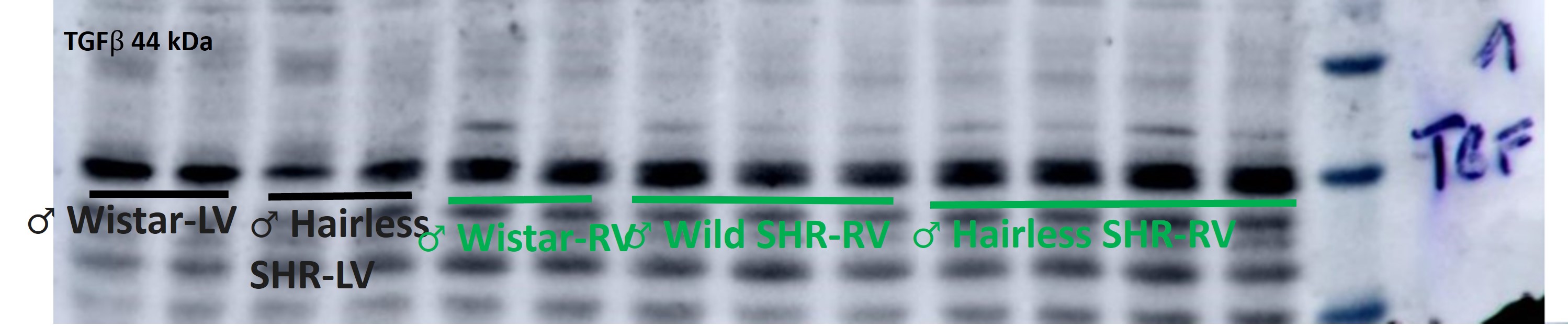

Supplement: Supplementary file 1 [file biomolecules-14-01509-s001.zip › TGFbeta MALE_membrane 3.jpg]
